# Traditional Medicinal Plants Used for Cancer Treatment in Sub-Saharan Africa: A Systematic Review

**DOI:** 10.3390/plants15121836

**Published:** 2026-06-13

**Authors:** Tomi Lois Adetunji, Funsho Oyetunde-Joshua, Olalekan Bukunmi Ogunro, Olumayowa Andrew, Stephen O. Amoo

**Affiliations:** 1 Agricultural Research Council—Vegetables, Industrial and Medicinal Plants, Private Bag X293, Roodeplaat, Pretoria 0001, South Africa; 2 Unit for Environmental Sciences and Management, Faculty of Natural and Agricultural Sciences, North-West University, Private Bag X6001, Potchefstroom 2520, South Africa; 3 Faculty of Natural and Environmental Sciences, Philomath University, Kuje 900105, Abuja, Federal Capital Territory, Nigeria; 4 Drug Discovery, Pharmacology and Toxicology Research Laboratory (DDPT-RL), Department of Biological Sciences, KolaDaisi University, Ibadan 200213, Oyo State, Nigeria; 5 Centre for Soil, Agrifood and Biosciences, Cranfield University, College Road, Bedfordshire MK43 0AL, UK

**Keywords:** anticancer activity, decoction, ethnobotany, ethnopharmacology, indigenous knowledge, phytochemistry

## Abstract

Cancer represents one of the major public health issues in sub-Saharan Africa (SSA), with increasing incidence and mortality rates as a result of late diagnosis, limited healthcare infrastructure, and financial difficulties. Traditional medicine plays an important role in healthcare across different populations in SSA, as more than 80% of the population depend on indigenous plant-based remedies for treating or managing different ailments, including cancer. This study aimed to document medicinal plants traditionally used to treat cancer in SSA. A systematic search of all documents available in the last two decades (2006–2026) was conducted using PubMed, Web of Science, and Google Scholar databases. After screening studies using the predefined inclusion and exclusion criteria, 55 studies met the eligibility requirements and were selected for analysis based on their relevance to the topic, geographic scope, and reported applications in cancer management. The scientific names of the identified plant species and their taxonomic authorities were verified using the Plants of the World Online database. A total of 556 species, belonging to 110 families, were recorded as medicinal plants used to treat various forms of cancer in SSA. The top five families with the most frequently used plants were Fabaceae (51 species), Asteraceae (34 species), Euphorbiaceae (25 species), Apocynaceae (22 species) and Lamiaceae (22 species). Frequently cited plants include *Kigelia africana*, *Annona muricata*, *Adansonia digitata*, *Carica papaya*, and *Tamarindus indica*. A total of 11 plant parts were documented, with leaves (41.20%), roots (18.75%), and bark (17.25%) being the dominant plant parts utilised. The primary methods of preparation were decoction (38.23%), powdering and grinding (14.51%), and infusion and tea preparation (49.73%), while the main modes of administration were oral (66.88%) and topical (26.46%). The results show that traditional medicinal plants hold significant potential as sources of novel anticancer drugs in SSA. However, a significant gap exists between ethnobotanical knowledge, laboratory research, and clinical application. Rigorous pharmacological and toxicity evaluations and well-designed clinical trials on the identified medicinal plants are needed to integrate effective and safe plant-based therapies into evidence-based oncology.

## 1. Introduction

Cancer represents a critical and escalating public health challenge globally, ranking as the second leading cause of mortality worldwide and the third leading cause of premature death in sub-Saharan Africa (SSA) [1]. The disease is characterised by the uncontrolled proliferation of abnormal cells that evade programmed cell death mechanisms. According to GLOBOCAN 2020 estimates, 19.3 million new cancer cases were diagnosed globally, resulting in approximately 10 million deaths [2]. Projections indicate a 70% increase in cancer incidence by 2030, driven by population ageing, lifestyle factors, environmental carcinogens, and tobacco and alcohol consumption, with the majority of this burden expected to occur in low- and middle-income countries (LMICs) where healthcare infrastructure remains inadequate [3,4].

Sub-Saharan Africa, comprising 48 countries with a current population exceeding one billion and projected to reach two billion by 2050, faces a disproportionate cancer-related morbidity and mortality burden. In 2020, the region recorded between 801,392 and 1,109,209 new cancer cases, with 520,158 to 711,429 associated deaths [1,5,6]. Cancer now accounts for approximately one in seven deaths in SSA, with breast and cervical cancers predominating among women, ranking first in 28 and 19 countries, respectively, while prostate, liver, and colorectal cancers are most prevalent among men [7,8,9]. The lifetime risk of developing breast cancer among women in SSA is estimated at 4.1%, with cervical cancer at 3.5%. Alarmingly, without urgent intervention, annual cancer mortality in the region is projected to nearly double from approximately 520,000 in 2020 to one million by 2030, reflecting an epidemiological transition toward non-communicable diseases [7,10].

The cancer burden in SSA is further exacerbated by systemic healthcare challenges. More than 70% of African cancer patients present at advanced stages of disease, significantly limiting curative treatment options [4]. The region has the lowest global availability of cancer diagnostic and therapeutic infrastructure, including severe shortages of oncology specialists, essential medicines, radiotherapy centres, and pathology services [9]. Financial barriers compound these challenges, as the high cost of conventional cancer therapies, including chemotherapy, surgery, radiotherapy, and immunotherapy, renders them inaccessible to the majority of the population. For instance, ethnographic surveys in Togo revealed that while tumours are diagnosed in hospitals, patients predominantly seek treatment from traditional healers using medicinal plants due to prohibitive oncology costs [11]. Furthermore, conventional therapies are associated with significant adverse effects, non-specific toxicity, and multidrug resistance mediated by drug efflux transporters, further limiting their clinical utility [12]. The concurrent use of traditional and complementary medicine (T&CM) among cancer patients undergoing conventional treatment is well documented in SSA, driven by accessibility, cultural acceptance, and perceived safety of herbal remedies [13].

Natural products have historically served as a rich source of anticancer therapeutics. Approximately 60% of currently approved anticancer drugs are derived from natural sources or are nature-inspired compounds [14,15,16]. Notable examples include vinblastine and vincristine from *Catharanthus roseus*, paclitaxel (Taxol) from *Taxus brevifolia*, topotecan from *Camptotheca acuminata*, and etoposide from *Podophyllum peltatum* [17]. Plant-derived phytochemicals exert anticancer effects through multiple mechanisms including induction of apoptosis, cell cycle arrest, inhibition of angiogenesis, and modulation of key tumorigenic signalling pathways [18]. The World Health Organization estimates that approximately 80% of the global population, particularly in developing regions, relies on traditional herbal medicine for primary healthcare [19]. In Africa, this dependency is particularly pronounced, with an estimated 80% of the population utilising medicinal plants owing to their accessibility, affordability, cultural acceptance, and perceived minimal side effects [20]. Africa’s diverse ecological zones including tropical rainforests, savannahs, and highland ecosystems, harbour extensive botanical diversity, providing a vast repository of medicinal flora. Traditional knowledge systems, transmitted intergenerationally, guide the therapeutic application of these plants, which are prepared through various methods including decoctions, infusions, and pastes among others. Phytochemical investigations have identified bioactive compounds in African medicinal plants, including alkaloids, flavonoids, terpenoids, and saponins, many of which exhibit cytotoxic activity against various cancer cell lines in vitro and in vivo [1,21].

Several reports have documented the ethnobotanical use of medicinal plants for cancer treatment in this region. For example, Raimi et al. [12] reported 207 plant species from 70 families, with 48% demonstrating documented cytotoxic activity, predominantly from the Asteraceae and Fabaceae families, targeting breast, skin, colorectal, cervical, and prostate cancers. A scientometric analysis by Adico et al. [3] identified 204 plant species across 57 families, with Amaryllidaceae, Fabaceae, Annonaceae, and Asteraceae being the most prevalent; plant extracts from these families have been shown to exert anticancer activity primarily through induction of apoptosis and cell cycle arrest [3]. Regional studies have revealed distinct ethnobotanical patterns: in East Africa, Kudamba et al. [22] documented 105 species from 45 families, with Uganda showing the highest diversity; in the Elgon Sub-Region of Uganda, 50 species from 36 families were identified, with Fabaceae and Asteraceae predominating and decoctions of leaves and roots representing the most common preparation methods [23,24]. Ethnobotanical surveys in central Uganda by Lutoti et al. [25] further documented the diversity of plants used by traditional health practitioners for breast cancer treatment, underscoring the depth of indigenous pharmacological knowledge. Similarly, in West Africa, ethnopharmacological surveys in Ghana’s Ashanti Region identified 151 plant species, with notable representation from the Meliaceae family [26], while studies conducted in Togo documented 70 species across 39 families, particularly Rubiaceae, Caesalpiniaceae, Fabaceae, and Annonaceae [11]. A comprehensive review by Coulibaly et al. [27] further characterised the molecular mechanisms of sub-Saharan African plant-derived substances against prostate and cervical cancer cells, providing a scientific basis for their ethnomedicinal use. Taken together, these findings highlight the enormous, untapped potential of SSA’s botanical heritage as a source of novel anticancer compounds, and underscore the urgent need for systematic phytochemical and pharmacological investigations to validate, optimise, and translate these traditional remedies into evidence-based therapeutic agents [28].

Despite these valuable contributions, a comprehensive and up-to-date systematic review synthesising ethnobotanical data on anticancer medicinal plants across the entire sub-Saharan African region remains lacking. This systematic review therefore aims to: (1) compile and analyse ethnobotanical data on medicinal plants traditionally used for cancer treatment in SSA; (2) document plant species, families, parts used, preparation methods, and cancer types addressed; (3) evaluate the phytochemical and pharmacological evidence supporting traditional uses; and (4) identify knowledge gaps and priorities for future research. By providing a consolidated evidence base, this review seeks to inform drug discovery initiatives, validate traditional knowledge systems, and contribute to the development of accessible, affordable, and culturally appropriate cancer therapeutics for sub-Saharan African populations.

## 2. Materials and Methods

### 2.1. Search Strategy

**Figure 1:**
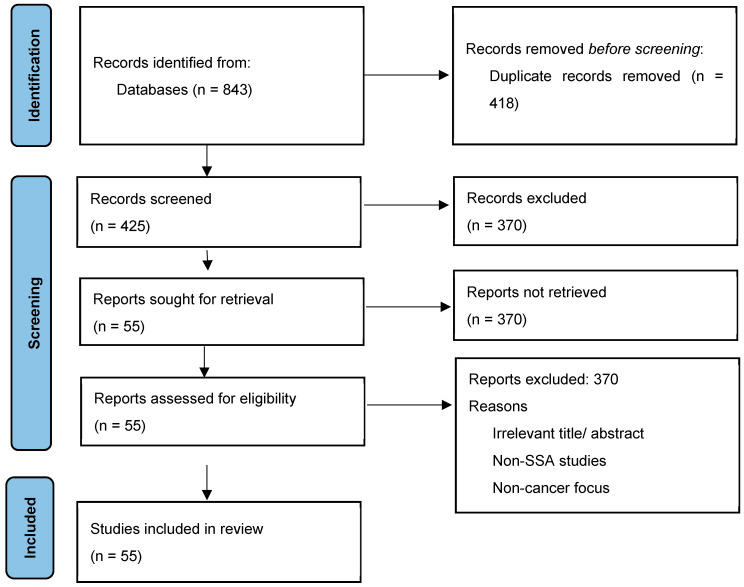
PRISMA 2020 flow diagram showing the systematic search and selection of studies on medicinal plants used for cancer management in sub-Saharan Africa.

A systematic literature search on medicinal plants used for cancer management in sub-Saharan Africa was conducted across three electronic databases: PubMed, Web of Science, and Google Scholar. The search covered all articles published from January 2006 to April 2026. The following keywords were used in the search: ethnobotany OR traditional medicine OR herbal medicine OR phytotherapy AND cancer OR anticancer OR antineoplastic OR tumour OR malignancy AND sub-Saharan Africa OR individual country names within the region AND medicinal plants OR ethnomedicine. Boolean operators and exact search strings were employed, such as (“medicinal plants” OR “ethnobotany” OR “traditional medicine” OR “herbal medicine”) AND (“cancer” OR “anticancer” OR “antineoplastic” OR “tumour” OR “malignancy” OR “oncology”) AND (“Sub-Saharan Africa” OR “West Africa” OR “East Africa” OR “Southern Africa” OR “Central Africa”). The search was restricted to articles published in English. In total, the initial search yielded 843 records from the databases. Following screening and eligibility assessment, 55 research articles met the predefined inclusion and exclusion criteria and were included in this review (Figure 1). Articles were initially screened based on title and abstract, after which full-text papers were retrieved and evaluated for eligibility. All keywords were searched electronically, while titles and abstracts were screened manually. This review was conducted in accordance with the PRISMA 2020 guidelines for reporting systematic reviews [29,30].

### 2.2. Inclusion and Exclusion Criteria and Data Extraction

Research articles were included if they described the use of medicinal plants for cancer treatment or management in sub-Saharan Africa and met the following inclusion criteria: (a) original research articles published in peer-reviewed journals; (b) ethnobotanical studies conducted in sub-Saharan African countries; (c) focus on medicinal plants traditionally used for cancer treatment or management; (d) provision of specific botanical identification using accepted scientific nomenclature for at least one plant species; and (e) articles written in English. Exclusion criteria included meta-analyses and studies focused exclusively on synthetic compounds or pharmaceutical derivatives; articles lacking detailed botanical information or species identification; research on non-cancer diseases or conditions and publications with unclear or poorly described methodologies. The scientific names of all identified plant species and their taxonomic authorities were validated using the Plants of the World Online (POWO) database to ensure nomenclatural accuracy and consistency. Data were extracted from eligible articles using a standardised form.

### 2.3. Quality Assessment

The methodological quality of included studies was assessed using adapted criteria for ethnobotanical research. Evaluation considered aspects such as proper botanical identification, documentation of traditional knowledge holders, ethical considerations, and clarity of reporting. No formal risk of bias assessment tool was applied.

## 3. Results

### 3.1. Geographical Distribution and Regional Variations

**Figure 2:**
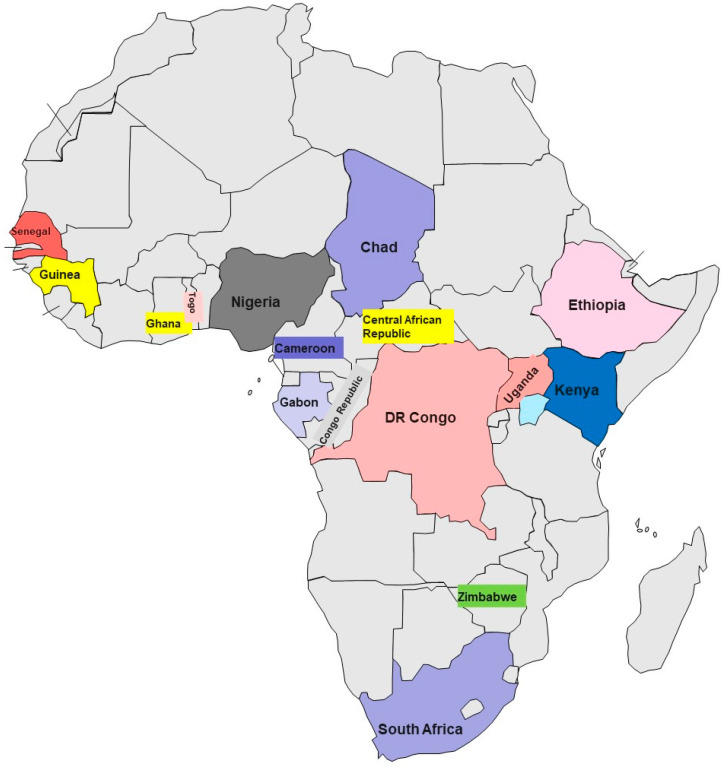
Geographical distribution of sub-Saharan African countries reporting traditional anticancer use of medicinal plants (n = 16 countries).

Figure 2 illustrates the geographical distribution of reported traditional anticancer medicinal plant use across sub-Saharan Africa. A total of 556 plant-use records were extracted from the included studies across sub-Saharan Africa, spanning West, East, Central, and Southern African sub-regions. Of the 16 countries represented in this review, the majority of plant-use records originated from West Africa, particularly Ghana (*n* = 170), Togo (*n* = 72), and Nigeria (*n* = 21), and East Africa, including Uganda (*n* = 74), Kenya (*n* = 37), and Ethiopia (*n* = 23), while Southern Africa contributed records from South Africa (*n* = 92) and Zimbabwe (*n* = 17). Central African countries were only marginally represented, with Cameroon (*n* = 11), Gabon (*n* = 1), the Republic of Congo (*n* = 1), the Democratic Republic of Congo (*n* = 1), Chad (*n* = 1), and the Central African Republic (*n* = 1) accounting for minimal documentation (Table 1). Notably, West African countries such as Senegal (*n* = 1) and Guinea (*n* = 1) were similarly under-represented (Table 1).

West Africa contributed the largest share of plant-use records within the dataset. Ghana alone accounted for 170 records representing 170 unique species, with almost all records derived from studies conducted in the Ashanti Region (*n* = 167), rendering it the single most extensively studied ethnic community in this review (Table 1). The dominant plant families documented in Ghana were Fabaceae (*n* = 14), Apocynaceae (*n* = 12), Euphorbiaceae (*n* = 11), and Malvaceae (*n* = 11). Ethnobotanical practice in the Ashanti community was characterised by a strong preference for decoction (*n* = 108) and tea preparation (*n* = 21), with oral administration constituting the predominant route of delivery. The most frequently reported cancer indications in Ghana were skin cancer (*n* = 85), breast cancer (*n* = 80), stomach cancer (*n* = 48), and prostate cancer (*n* = 38). Togo contributed 72 records representing 72 unique species, with plant use reported predominantly among the Kabyè (*n* = 25) and Tem (*n* = 20) ethnic communities. The leading families in Togo were Caesalpiniaceae, Fabaceae, and Rubiaceae (each *n* = 5), and Annonaceae and Meliaceae (each *n* = 3). Preparation in Togo was characterised by a dual dominance of decoction (*n* = 34) and powdering (*n* = 34), alongside notable use of medicinal sauces (*n* = 12), a culturally distinctive preparation method not prominently recorded elsewhere in the dataset. The primary cancer indications reported in Togo were breast cancer (*n* = 32), chronic wound and ulcerous conditions (*n* = 28), and lung cancer (*n* = 17). Nigeria contributed 18 records across 21 unique species, with studies from Adamawa State (*n* = 8) documenting use among the Mubi community (Table 1).

East Africa was represented primarily by Uganda and Kenya. Uganda yielded 74 plant-use records spanning 71 unique species, documented predominantly within the Elgon Sub-Region (*n* = 47) and Kampala (*n* = 6), with additional records from Mukono/Buikwe (*n* = 4) and Pallisa (*n* = 2). The leading families in Uganda were Fabaceae (*n* = 8), Asteraceae (*n* = 5), Lamiaceae and Sapindaceae (each *n* = 3). Preparation in Uganda was dominated by decoction (*n* = 38), alongside burning of plant materials (*n* = 9) and boiling (*n* = 9), practices less commonly documented in West African studies. The most frequently targeted cancers in Uganda were prostate (*n* = 32) and cervical cancers (*n* = 32), followed by lung cancer (*n* = 22) and gastrointestinal tract (GIT) cancers (*n* = 19), indicating a distinctly different cancer-treatment profile compared to West African communities. Ethiopia contributed 23 records spanning 25 unique species, with decoction also predominating as the principal preparation method. Kenya yielded 37 records representing 38 unique species, with the most commonly addressed cancers being colorectal (*n* = 16), breast (*n* = 15), and skin (*n* = 12) cancers.

Southern Africa was represented principally by South Africa (*n* = 92 records) and Zimbabwe (*n* = 17 records; 17 unique species). South Africa exhibited the most diverse botanical family representation in this sub-region, with Asteraceae (*n* = 13), Lamiaceae (*n* = 8), Hypoxidaceae (*n* = 5), Amaryllidaceae (*n* = 4), and Apocynaceae (*n* = 4) being most prominent. Notably, the South African studies showed a higher proportion of laboratory-validated cancer targets, with several records referencing specific cancer cell lines including MCF-7 (breast), CCRF-CEM and CEM/ADR5000 (leukaemia), DU-145 (prostate), and renal TK10 and melanoma UACC62 cell lines, reflecting a more pharmacologically oriented reporting approach compared to purely ethnobotanical documentation observed in West and East African studies. Studies in South Africa were conducted among the Eastern Cape and Western communities. Zimbabwe contributed records across the Bulawayo (*n* = 2), Manicaland (*n* = 2), and Masvingo (*n* = 2) provinces (Table 1).

Several plant species were documented across more than one country or sub-region, indicating widespread ethnomedicinal utilisation and potential cross-cultural transmission of traditional knowledge. Conversely, a number of species appeared geographically restricted, likely reflecting local ecological availability, endemic botanical distribution, or culturally specific healing traditions. Overall, the geographic data reveal distinct regional variations not only in species selection and family diversity, but also in cancer-type priorities, preparation methods, and the depth of ethnobotanical documentation (Table 1).

### 3.2. Diversity of Medicinal Plant Species and Families

**Figure 3:**
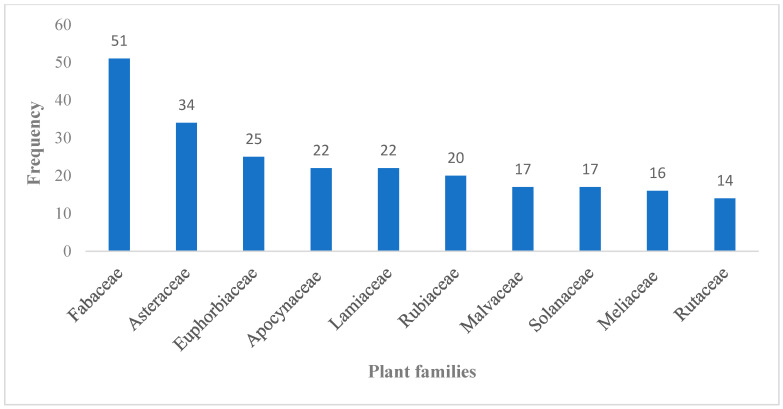
Top 10 plant families with the most species used in cancer treatment in Sub-Saharan Africa.

The compiled data presented in Table 1 revealed a broad diversity of medicinal plants traditionally used for cancer management across sub-Saharan Africa. A total of 556 plant species belonging to 110 botanical families were documented across the included studies. Several plant families were consistently represented across multiple studies, reflecting their prominence in traditional anticancer medicine in the region. The top five families with the highest species representation were Fabaceae (*n* = 51), Asteraceae (*n* = 34), Euphorbiaceae (*n* = 25), Apocynaceae (*n* = 22), and Lamiaceae (*n* = 22). Other frequently represented families included Rubiaceae (*n* = 20), Malvaceae (*n* = 17), Solanaceae (*n* = 17), Meliaceae (*n* = 16), and Rutaceae (*n* = 14) (Figure 3). The consistent representation of Fabaceae and Asteraceae across multiple geographically distinct studies highlights their particular significance in indigenous cancer-related ethnomedicine throughout the region.

Among the most frequently cited individual species, *Kigelia africana* (Lam.) Benth. was the most recurrently documented species, recorded across five independent studies. This was followed by *Annona muricata* L., *Adansonia digitata* L., *Carica papaya* L., *Tamarindus indica* L., and *Moringa oleifera* Lam., each cited in three studies. The recurrence of these species across geographically distinct study populations suggests widespread and cross-culturally consistent traditional knowledge regarding their anticancer properties.

### 3.3. Plant Parts Utilised

**Figure 4:**
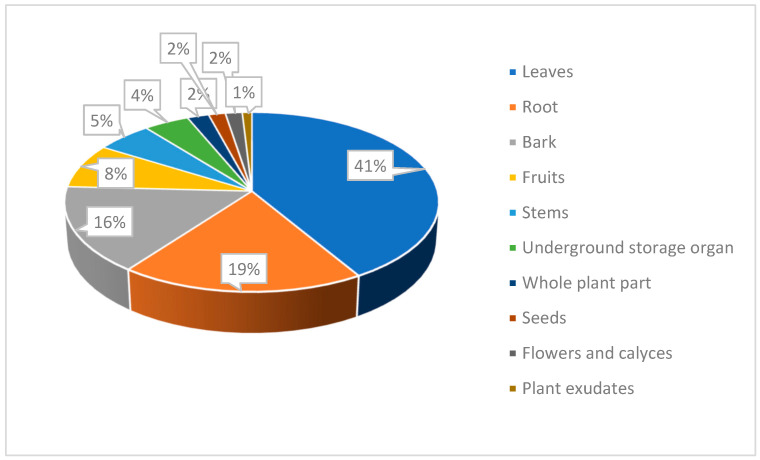
Distribution of plant parts used in cancer treatment in Sub-Saharan Africa.

A total of 11 distinct plant part categories were documented across the included studies, with a total of 864 individual plant part mentions recorded. Leaves were the most predominantly utilised plant part, accounting for 356 mentions (41.20%), followed by roots (n = 162; 18.75%), and bark, including stem bark and root bark (*n* = 134; 15.51%). Fruits represented the fourth most commonly documented plant part (*n* = 69; 7.99%), followed by stems (*n* = 47; 5.44%), underground storage organs including rhizomes, bulbs, corms, and tubers (*n* = 38; 4.39%), and the whole plant (*n* = 18; 2.08%). Less frequently documented plant parts included seeds (*n* = 14; 1.62%), flowers and calyces (*n* = 14; 1.62%), and plant exudates including latex, sap, and resin (*n* = 8; 0.93%) (Figure 4).

The predominance of aerial vegetative parts, particularly leaves, is consistent with patterns reported in ethnobotanical literature across sub-Saharan Africa, where leaves are generally more accessible, renewable, and less destructive to harvest than underground organs.

### 3.4. Methods of Preparation

Across the 586 individual preparation methods recorded, decoction was by far the most frequently employed method, accounting for 224 mentions (38.23%). This was followed by powdering and grinding (*n* = 85; 14.51%), infusion and tea preparation (*n* = 57; 9.73%), paste and poultice preparation (*n* = 23; 3.92%), boiling by methods other than decoction (*n* = 22; 3.75%), juice extraction and squeezing (*n* = 21; 3.58%), maceration (n = 18; 3.07%), and raw consumption (*n* = 9; 1.54%) (Table 1). The dominance of decoction as the primary mode of preparation is consistent with the broader ethnobotanical literature and likely reflects the efficiency of aqueous extraction in rendering bioactive phytochemical constituents bioavailable.

### 3.5. Routes of Administration

Five principal routes of administration were recorded across the 480 individual administration mentions documented in Table 1. Oral administration was overwhelmingly predominant, accounting for 321 mentions (66.88%), followed by topical application (*n* = 127; 26.46%). Less common routes included body bathing (*n* = 9; 1.88%), massage (*n* = 9; 1.88%), nasal administration (*n* = 5; 1.04%), and inhalation or smoking (*n* = 2; 0.42%). Oral and topical routes combined accounted for over 93% of all recorded administration modes, reflecting the principal delivery mechanisms employed in traditional cancer management within the region.

### 3.6. Cancer Types Addressed

**Figure 5:**
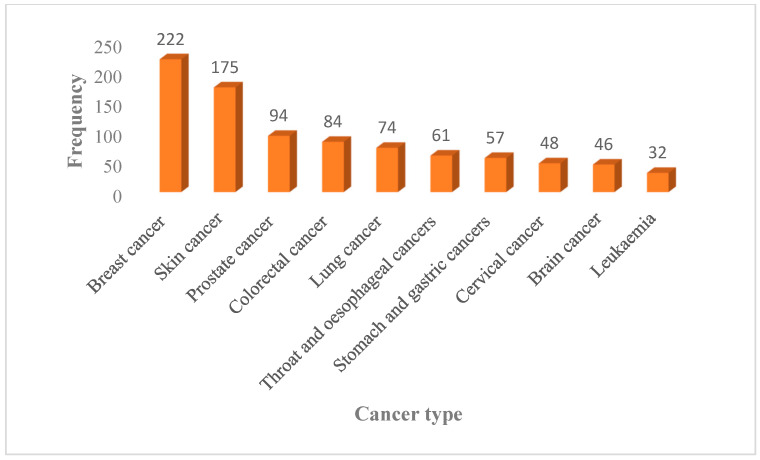
Top 10 cancers most frequently treated by medicinal plants in Sub-Saharan Africa.

The documented medicinal plants were reported for use against a diverse range of cancer types (Figure 5). Breast cancer was the most frequently targeted malignancy, accounting for 222 plant-use records (24.3% of all cancer-type mentions), followed by skin cancer and melanoma (*n* = 175; 19.2%), prostate cancer (*n* = 94; 10.3%), colorectal cancer (*n* = 84; 9.2%), and lung cancer (*n* = 74; 8.1%). Additional cancer types reported included throat and oesophageal cancers (*n* = 61; 6.7%), stomach and gastric cancers (*n* = 57; 6.2%), cervical cancer (*n* = 48; 5.3%), brain cancer (*n* = 46; 5.0%), leukaemia (*n* = 32; 3.5%), and liver and hepatic cancers (*n* = 20; 2.2%). The preponderance of breast and skin cancer records is consistent with epidemiological data indicating the high burden of these malignancies among women in sub-Saharan Africa, and with the accessibility of topically applied plant preparations for visible or surface tumours.

### 3.7. Molecular Mechanism of Anticancer Action of Selected Plant Species

While the vast majority of plant species documented in this review are supported solely by ethnobotanical evidence, some of the identified species have been subjected to experimental pharmacological investigation to elucidate their molecular mechanisms of anticancer action. This section summarises the available mechanistic evidence for six priority species that are either among the most frequently cited in Table 1, are already the source of clinically approved anticancer drugs, or have been validated against cancer cell lines. These mechanistic findings provide an evidence base to contextualise traditional use and identify lead compounds for further pharmaceutical development.

***Catharanthus roseus*** **(L.) G.Don**

*Catharanthus roseus*, documented in this review from South Africa and Uganda (Table 1), is the source of two of the most clinically important anticancer agents of plant origin: vincristine and vinblastine. These bisindole alkaloids exert their cytotoxic effects by binding to β-tubulin at the vinca domain, thereby inhibiting tubulin polymerisation and blocking assembly of the mitotic spindle [38]. This binding causes metaphase arrest by preventing the dynamic instability of microtubules required for chromosome segregation, ultimately triggering apoptosis in rapidly proliferating cancer cells [38]. Molecular docking analyses have identified key interaction sites including C-Lys336 and B-Glu183 residues on β-tubulin, with both catharanthine and vindoline moieties of the dimeric alkaloids contributing to binding affinity [38]. Vincristine is currently used clinically for the treatment of acute leukaemia, Hodgkin’s lymphoma, and paediatric solid tumours, while vinblastine is used for the treatment of testicular cancer and Hodgkin’s disease. Multidrug resistance mediated by P-glycoprotein (MDR1/ABCB1) overexpression is the principal mechanism by which cancer cells acquire resistance to vinca alkaloids, providing a rationale for exploring combinatorial or structural analogue strategies to overcome resistance [38].

***Annona muricata*** **L.**

*Annona muricata* (soursop), documented across Ghana, Togo, and Uganda in this review, contains annonaceous acetogenins (ACGs) as its principal bioactive class. These long-chain fatty acid derivatives, including bullatacin, annomuricin A, annohexocin, and muricatocin A, are among the most potent natural cytotoxins identified, with IC_50_ values in the nanomolar range against multiple cancer cell lines [39]. ACGs exert anticancer effects primarily through selective inhibition of mitochondrial NADH:ubiquinone oxidoreductase (Complex I), resulting in ATP depletion, collapse of mitochondrial membrane potential, and induction of the intrinsic apoptotic pathway [39]. Downstream consequences include increased Bax/Bcl-2 ratio, cytochrome c release into the cytosol, and sequential activation of caspase-9 and caspase-3 [39]. Additional mechanisms include cell cycle arrest at the G_0_/G_1_ phase and suppression of NF-κB translocation from cytoplasm to nucleus, thereby inhibiting pro-survival gene transcription in lung (A549), breast (MCF-7), colon, and prostate cancer cell lines [40]. Molecular docking studies have demonstrated stable interactions of ACGs with Bcl-2, Bcl-xL, and Mcl-1 anti-apoptotic proteins, reinforcing the multi-target nature of this compound class [41]. The selective toxicity of ACGs toward cancer cells over normal cells is attributed to the higher energy demands and altered mitochondrial activity of malignant cells.

***Kigelia africana*** **(Lam.) Benth.**

*Kigelia africana*, the most frequently cited species in this review (documented across five independent studies from Togo, Uganda, Ghana, Zimbabwe, and South Africa), has been the subject of multiple pharmacological investigations. The fruit and bark contain naphthoquinones (kigelinole, isokigelinole, pinnatal, isopinnatal, lapachol), iridoid glycosides (specioside, verminoside, minecoside, catalpol), phenolic acids, and ellagitannins [42]. Mechanistic studies in HCT116 colorectal cancer cells demonstrated that *K. africana* fruit extract modulated Bax/Bcl-2 expression in a dose-dependent manner, activating caspase-3, caspase-9, and cleaved PARP (poly-ADP-ribose polymerase), which are hallmarks of the intrinsic mitochondrial apoptotic pathway [42]. Cytometric analysis revealed sub-G_1_ phase accumulation indicative of apoptosis, alongside activation of the MAPK signalling pathway [42]. A recent integrative evaluation in HT-29 colorectal carcinoma cells demonstrated that *K. africana* fruit extract suppressed 42 oncology-related proteins across cell survival, apoptosis, adhesion, invasion, and signalling networks, and produced marked synergistic cytotoxicity in combination with cisplatin [43]. Studies in MDA-MB-231 and MCF-7 breast cancer cells further showed modulation of TP53 expression and inhibition of HER2-linked signalling cascades [43]. The selective cytotoxicity of *K. africana* extracts towards malignant cells and not on normal cells suggests specific modulation of defined molecular tumour targets and supports its prioritisation as a candidate for bioassay-guided fractionation and lead compound identification or for standardised active ingredient development.

***Moringa oleifera*** **Lam.**

*Moringa oleifera*, documented across Zimbabwe, Togo, and Uganda, is rich in glucosinolates, principally glucomoringin, whose enzymatic hydrolysis by myrosinase yields the isothiocyanate 4-[(α-L-rhamnosyloxy)benzyl] isothiocyanate (moringin; MIC-1) [44]. This bioactive compound has been demonstrated to inhibit proliferation of human neuroblastoma (SH-SY5Y), glioma (U251), breast (MDA-MB-231), cervical (HeLa), and renal (786-O) cancer cell lines through different mechanisms [44]. In glioma cells, MIC-1 derivatives significantly increased the Bax:Bcl-2 ratio and activated caspase-3, arresting cells at the G_2_/M phase [44]. In A549 non-small-cell lung cancer cells, alkaloid extract of *M. oleifera* suppressed JAK2/STAT3 signalling, a pathway critically involved in tumour cell proliferation, survival, and immune evasion [45]. In renal carcinoma, MIC-1 inhibited cell migration and invasion by suppressing PTP1B-dependent Src/Ras/Raf/ERK signalling and reducing expression of matrix metalloproteinases MMP-2 and MMP-9 [46]. In lymphoma models, *M. oleifera* leaf extract induced G_2_/M phase arrest, upregulated p53 and p21, and activated MEK/ERK-mediated apoptotic pathways in vivo [46]. The isothiocyanate pharmacophore (R–NCS) also directly targets IKKβ (IκB kinase beta), suppressing NF-κB activation and downstream pro-inflammatory and pro-survival gene transcription [46], providing a mechanistic basis for the broad anticancer activity reported across multiple cancer types in ethnobotanical surveys.

***Vernonia amygdalina*** **Delile**

*Vernonia amygdalina* (bitter leaf), documented for general cancer use in Ethiopia, is a member of the Asteraceae family with a rich sesquiterpene lactone chemistry. Key bioactive compounds include vernodalin, vernolepin, vernolide, vernodalol, and vernodalidimer [47]. These elemanolide sesquiterpenoids have demonstrated potent, broad-spectrum cytotoxicity against multiple cancer cell lines, with vernolide showing IC_50_ values of 0.91–13.84 μM across ten cancer lines [47]. Mechanistically, vernodalin, vernolepin, and vernolide induce G_2_/M phase cell cycle arrest and dose-dependent apoptosis in HepG2 hepatocellular carcinoma cells, with annexin-V flow cytometry confirming phosphatidylserine externalisation as a hallmark of early apoptosis [47]. In A549 lung cancer cells, these compounds arrested the cell cycle at G_0_/G_1_ at sub-cytotoxic doses and at G_2_/M at cytotoxic doses, with mechanistic studies confirming modulation of the JAK2/STAT3 pathway via hydrogen-bond interactions at the FERM domain of JAK2 identified by molecular docking [47]. Anti-proliferative and anti-metastatic effects are further mediated by targeting ERK-1, ERK-2, NF-κB, STAT3, MMP-2, and MMP-9 [48]. At the apoptotic level, caspase-9 and caspase-3 activation is enhanced while Bcl-2 and Bcl-xL expression is inhibited, triggering cytochrome c release consistent with the intrinsic pathway. Whole leaf extracts of *V. amygdalina* also downregulate the PI3K/AKT/mTOR pathway and suppress HER2-mediated signalling in breast cancer models, providing multiple molecular entry points for anticancer activity [47].

***Hypoxis hemerocallidea*** **Fisch. & C.A.Mey**

*Hypoxis hemerocallidea* (African potato), documented in South Africa, Zimbabwe, and Uganda in this review, and explicitly referenced against DU-145 (prostate), MDA-MB-231 and MCF-7 (breast), MCF7, renal TK10, and melanoma UACC62 cell lines in Table 1, contains the bis-glucoside hypoxoside as its principal bioactive compound [48]. Hypoxoside itself is non-toxic but acts as a natural prodrug: upon oral ingestion, it is hydrolysed by intestinal β-glucosidase to release rooperol, a catechol-containing aglycone with potent cytotoxic activity [48]. Rooperol has been demonstrated to induce cell cycle arrest at the late G_1_/early S-phase transition in HeLa (cervical), HT-29 (colorectal), and MCF-7 (breast) cancer cell lines, associated with increased p21protein levels [48]. Apoptosis was confirmed by caspase-3 and caspase-7 activation, phosphatidylserine translocation (annexin V assay), DNA fragmentation, cell blebbing, and apoptotic body formation [48]. In MCF-7 cells, which lack functional caspase-3, rooperol selectively activated the caspase-7 compensatory pathway. Mechanistic studies further demonstrated that rooperol modulates pAkt and pBcl-2 phosphorylation, positioning it at the PI3K/Akt/Bcl-2 node as a survival pathway disruptor [48].

Taken together, these mechanistic findings across six species documented in Table 1 identify five core mechanisms through which SSA medicinal plants exert anticancer activity: (1) microtubule disruption and mitotic arrest; (2) mitochondrial complex I inhibition and intrinsic apoptosis; (3) modulation of Bcl-2 family proteins and caspase cascades; (4) inhibition of JAK2/STAT3, NF-κB, ERK, and PI3K/Akt survival signalling pathways; and (5) cell cycle arrest at G_1_/S or G_2_/M checkpoints mediated by p21 and p53 upregulation. These mechanisms collectively align with recognised hallmarks of cancer and reinforce the pharmacological basis for prioritising the experimental investigation of the many ethnobotanically documented species that remain mechanistically uncharacterised.

## 4. Discussion

This systematic review brings together ethnobotanical evidence on medicinal plants used for cancer management across sub-Saharan Africa, based on data systematically extracted and summarised in Table 1. Use of PRISMA 2020 guidelines provided a transparent framework for study identification, selection, and synthesis [30]. The findings reflect the continued importance of traditional medicine in cancer care, particularly in settings where access to specialised oncology services remains limited or inconsistent [49,50]. A total of 556 plant species distributed across 110 botanical families were documented across 16 sub-Saharan African countries, representing one of the more comprehensive ethnobotanical compilations of anticancer plant use in the region to date. The breadth of species and families recorded across geographically and ecologically diverse settings underscores the richness of indigenous botanical knowledge systems and their enduring role in community-level cancer management.

The range of medicinal plants documented in this review highlights the depth of traditional medical knowledge across the region. Several plant families, most notably Fabaceae, Asteraceae, Apocynaceae, Euphorbiaceae, Rubiaceae, Amaryllidaceae, Anacardiaceae, and Lamiaceae, were repeatedly reported. Similar patterns have been observed in earlier African ethnopharmacological surveys [51,52]. These families are well known for producing secondary metabolites such as alkaloids, flavonoids, terpenoids, and phenolic compounds, many of which have demonstrated cytotoxic or growth-inhibitory effects in cancer models [53,54]. Among individual species, *Kigelia africana* (Lam.) Benth. was the most frequently cited across five independent studies, followed by *Annona muricata* L., *Adansonia digitata* L., *Carica papaya* L., *Tamarindus indica* L., and *Moringa oleifera* Lam., each cited in three studies. The recurrence of these species across geographically distinct study populations is particularly noteworthy, as it suggests a degree of cross-cultural consensus regarding their perceived therapeutic value that transcends regional boundaries. Pharmacological investigations have confirmed the cytotoxic and antiproliferative activity of *K. africana* extracts against multiple cancer cell lines, including melanoma, breast cancer, and renal carcinoma cells [42], while *A. muricata* has been shown to contain annonaceous acetogenins with potent anticancer activity against breast, prostate, colon, and liver cancer cell lines [39]. Such convergence of independent traditional knowledge systems may strengthen the ethnopharmacological rationale for prioritising these species in future laboratory and clinical investigations.

Reports from West, East, Central, and Southern Africa point to both ecological diversity and long-standing exchange of medicinal knowledge between communities. The strong representation of West African countries may partly reflect greater research activity in these areas, alongside well-established traditions of herbal medicine use [55]. Ghana alone accounted for the largest share of plant-use records (n = 170), with all records derived from studies conducted in the Ashanti Region, suggesting that this community represents a particularly rich repository of ethnomedicinal knowledge that warrants further investigation. The appearance of the same plant species in different countries suggests shared therapeutic knowledge, while species confined to specific regions likely reflect local plant availability and culturally distinct practices. Notably, a substantial geographic imbalance was observed in the distribution of included studies, with Central African countries, island nations of the Indian Ocean, and several Horn of Africa countries entirely absent from the reviewed literature. Given that sub-Saharan Africa comprises 49 countries, the representation of only 16 in this review signals a considerable research gap that cannot be attributed solely to the absence of traditional plant use in unrepresented regions, but more likely reflects limited ethnobotanical research activity and publication in these areas.

Leaves were the most frequently used plant part (41.20%), followed by roots (18.75%) and bark (15.51%). This pattern is consistent with global ethnobotanical observations [56]. Leaves are generally easier to harvest and regenerate quickly, and they are metabolically active tissues rich in secondary metabolites available for extraction via decoction and infusion [57]. Fruits, stems, and bulbs, corms, and tubers collectively accounted for a further 17.25% of documented plant part use, while flowers, whole plant preparations, latex, and rhizomes were less commonly recorded. In contrast, the reliance on roots and bark, particularly from woody species, reflects traditional views of potency but also raises concerns. Harvesting these parts can be destructive and has been associated with reduced plant populations when carried out unsustainably [58]. The considerable proportion of root and bark use documented in this review, together accounting for more than a third of all plant part mentions, therefore warrants particular attention from conservation and sustainable harvesting perspectives, especially for species identified across multiple studies. Conservation and propagation strategies for threatened medicinal plant species are critically needed across the region to ensure their continued availability for both traditional use and scientific investigation [59].

Decoction emerged as the dominant method of preparation (38.23%), followed by powdering and grinding (14.51%), and infusion and tea preparation (9.73%). This approach facilitates extraction of water-soluble constituents and may enhance availability of active compounds [60]. Other preparation methods, including infusions, powders, and topical pastes, were also reported, indicating flexibility in formulation across different communities and ecological settings. Polyherbal remedies were documented in several studies, reflecting traditional practices that combine multiple species within a single preparation. Such combinations are increasingly recognised in phytotherapy research for their potential to produce complementary or additive effects [53,61]. Recent experimental evidence further supports the synergistic anticancer potential of herbal combinations, with multiple studies reporting enhanced cytotoxicity, apoptosis induction, and tumour growth inhibition when herbal preparations are used in combination compared to single-agent formulations [62].

Oral administration was the most commonly reported route (66.88%), suggesting that traditional remedies are primarily intended to act systemically. This observation aligns with findings from other African ethnopharmacological studies [41,54]. Topical application was the second most frequently documented route (26.46%) and was frequently associated with skin cancers and externally visible tumours, allowing for direct contact between plant preparations and affected tissues. Together, oral and topical routes accounted for over 93% of all documented administration modes, with massage, body bathing, nasal administration, and inhalation or smoking constituting the remaining minority. In some cases, remedies were administered both orally and topically, reflecting integrated treatment approaches within traditional medical systems [52,63,64]. The medicinal plants documented were used for a wide range of cancer types, with breast cancer (24.3%), skin cancer and melanoma (19.2%), prostate cancer (10.3%), colorectal cancer (9.2%), and lung cancer (8.1%) most frequently reported, followed by throat and oesophageal, stomach, cervical, brain, leukaemia, and liver cancers. This may partly reflect disease prevalence, but also the visibility of symptoms and the diagnostic frameworks used in traditional medicine [65]. The preponderance of breast and skin cancer records is broadly consistent with the epidemiological burden of these malignancies in sub-Saharan Africa, and with the accessibility of topically applied preparations for surface or visible tumours. The relatively high frequency of prostate cancer documentation, particularly in Ugandan studies, aligns with the prominence of this malignancy among men in East Africa. Several species were reported for multiple cancer types, suggesting perceived broad-spectrum activity. These claims remain largely untested and highlight the need for systematic pharmacological evaluation [54]. Furthermore, the distinct cancer-type profiles observed across different countries with Ghana recording the highest number of skin and breast cancer entries while Uganda showed greater documentation of prostate and cervical cancers, reflect not only regional disease burden differences but also the influence of community-specific healing traditions and the scope of individual studies.

Only a limited number of the documented plants have been investigated experimentally, mainly through in vitro studies. A notable exception was observed in the South African literature, where several plant-use records referenced specific cancer cell lines including MCF-7 (breast), CCRF-CEM and CEM/ADR5000 (leukaemia), DU-145 (prostate), and renal TK10 and melanoma UACC62, indicating that some South African studies incorporated pharmacological validation alongside ethnobotanical documentation, a more integrated approach that other sub-regional research communities may benefit from adopting. Most species across the broader dataset, however, remain supported solely by ethnobotanical reports. Similar gaps have been noted in previous reviews of African medicinal plants used in cancer management [12]. The translation of ethnobotanical knowledge into evidence-based therapeutics demands a rigorous pipeline encompassing bioassay-guided fractionation, structure elucidation of active constituents, mechanistic studies in relevant cancer models, and preclinical toxicology [16]. Advancing this field will require carefully designed in vitro and in vivo studies, followed by toxicological assessments and, where appropriate, clinical investigation [51].

The clustering of traditional anticancer use within certain plant families points to clear opportunities for natural product research and lead compound identification [54]. At the same time, the frequent use of roots and bark highlights the importance of conservation, cultivation, and sustainable harvesting strategies. Protecting plant biodiversity is closely linked to preserving the traditional knowledge systems that underpin these practices [66]. The data gaps identified in this review particularly with respect to preparation methods, routes of administration, and geographic coverage further highlight the need for standardised ethnobotanical reporting frameworks and expanded primary research across currently underrepresented sub-Saharan African countries. The establishment of harmonised reporting standards across African ethnobotanical studies would substantially enhance the comparability and utility of future data for pharmacological prioritisation [67].

The toxicity profile and active concentration range of compounds exhibiting anticancer potential in plants are indispensable parameters for drug development. The assumption that plant-derived remedies are inherently safe by virtue of their traditional use is a widely recognised misconception that can have serious clinical implications [13]. For example, vincristine and vinblastine from *Catharanthus roseus* have been reported to have clinically significant adverse effects. Vincristine-induced peripheral neuropathy (VIPN) is among the most common and clinically significant adverse effects, affecting sensory, motor, and autonomic nerve function; symptoms can appear within one week of initiation of therapy and may persist for years beyond treatment conclusion [68]. The severity of VIPN is dose-dependent and represents a leading cause of vincristine dose reduction, delay, or discontinuation, which is a particularly important consideration given the central role of this alkaloid in paediatric oncology regimens [68]. These dose-limiting toxicities underline the profound clinical gap between the controlled intravenous administration of purified vinca alkaloids and the unregulated oral and topical preparations of *C. roseus* documented across South Africa and Uganda in this review. In contrast, *Kigelia africana* fruit and stem bark extracts have demonstrated a more encouraging profile, with IC_50_ values ranging near and well below 4–30 µg/mL against multiple cancer cell lines in a dose-dependent manner, and a cytotoxicity profile highly biased toward malignantly transformed rather than normal cells, suggesting specific modulation of defined molecular tumour targets [69]. Similarly, aqueous leaf extract of *Vernonia amygdalina* exhibited dose-dependent cytotoxicity against Jurkat, MCF-7, HepG2, and PNT2 cell lines, with an IC_50_ of 96.3 µg/mL against Jurkat cells and a selectivity index of 3.567 [70]. Regarding *Moringa oleifera*, a 13-week repeated oral dosing study in mice demonstrated that the optimised aqueous leaf extract did not affect physiological or haematological parameters at 250 and 500 mg/kg, attributing low or absent toxicity at these doses; however, the highest dose tested of 1000 mg/kg produced elevated transaminases and histopathological changes consistent with hepatic damage, indicating that a safety ceiling exists and that dose selection is critical [71]. The most serious safety concern among the frequently cited species in this review relates to *Annona muricata*. Its principal bioactive class, the annonaceous acetogenins, has been causally linked to atypical Parkinsonism through chronic dietary exposure; the EFSA risk assessment concluded that substantial uncertainties exist regarding the safe use of *A. muricata*-based preparations, that data provide strong indications of neurotoxic potential, and that the available evidence does not currently allow for the establishment of a safe intake level [72]. This finding has profound implications for the populations in Ghana, Togo, Uganda, and Ethiopia documented in this review as using *A. muricata* orally for a range of cancers, and represents a critical safety signal that must be investigated before any clinical translation is pursued. *Hypoxis hemerocallidea*, cited against DU-145, MCF-7, and renal TK10 cancer cell lines in Table 1, presents a distinct safety challenge through pharmacokinetic interactions. Aqueous corm extracts inhibited CYP1A2, CYP2B6, CYP2C9, and CYP3A4/5, as well as P-glycoprotein transport; the authors noted that extract concentrations exceeding the measured IC_50_ values are achievable in the gastrointestinal tract at traditional doses, meaning that presystemic and potentially systemic inhibition of drug metabolism is a realistic clinical concern [73]. Given the high HIV/AIDS burden across SSA and the documented co-use of this plant with antiretroviral therapy, this interaction profile represents a clinically significant risk in the precise patient populations where the plant is most widely used. Collectively, these findings reinforce that the phytochemical characterisation and bioactivity documentation of SSA anticancer plants must be accompanied by systematic profiling against normal cell lines, rigorous selectivity index determination, and comprehensive in vivo toxicological evaluation before any of these plant-derived agents can be responsibly advanced toward clinical development.

Beyond toxicity, a further consideration of fundamental importance to pharmaceutical research is the phytochemical complexity of the plant materials documented in this review. A single medicinal plant species may contain several hundred structurally distinct secondary metabolites, and the concentration of biologically active constituents is not fixed. It varies substantially with soil composition, climate, altitude, and time of collection [16,74]. The species most frequently cited in this review, including *Kigelia africana*, *Annona muricata*, and *Moringa oleifera*, each contain complex multicomponent phytochemical profiles whose composition shifts across geographic populations and seasons [74,75]. Testing such crude multicomponent preparations, which constitute the majority of the documented traditional medicines in Table 1 is inherently more complex than studying a pure compound, and raw extract IC_50_ data cannot be directly equated with the activity of any single constituent [53]. Bioassay-guided fractionation, followed by structural elucidation of isolated actives and validation of their concentrations against internationally recognised collection and standardisation frameworks such as the WHO Good Agricultural and Collection Practices (GACP) guidelines, is therefore an indispensable prerequisite before any of the plant species documented in this review can be advanced along a rigorous drug development pathway [53,76].

Taken together, the findings presented in this study and the accompanying analyses provide a structured overview of traditional anticancer plant use across sub-Saharan Africa. The patterns identified here offer a practical starting point for future pharmacological work while reinforcing the continued relevance of ethnobotanical knowledge in phytomedicine research. The convergence of species documentation across independent studies, the dominance of a small number of plant families, and the clear regional variations in both species selection and cancer-type focus collectively suggest that this body of traditional knowledge, despite its current fragmentation, holds significant and as yet largely untapped potential for informing the development of novel, accessible, and culturally grounded anticancer therapeutics for sub-Saharan African populations.

## 5. Conclusions

This systematic review provides a comprehensive synthesis of ethnobotanical evidence on medicinal plants traditionally used for cancer management across sub-Saharan Africa, documenting 556 plant species belonging to 110 botanical families from 16 countries. The findings confirm that traditional plant-based cancer therapy remains deeply embedded in healthcare practices across the region, particularly in communities where access to conventional oncology services is limited. The consistent documentation of the same plant families, notably Fabaceae, Asteraceae, Euphorbiaceae, Apocynaceae, and Lamiaceae, across geographically distinct and culturally diverse populations suggests a convergence of indigenous botanical knowledge that lends ethnopharmacological credibility to these plant groups as priority candidates for further scientific investigation. Within these families, species with the highest cross-cultural documentation, including *Kigelia africana*, *Annona muricata*, *Moringa oleifera*, *Carica papaya*, and *Adansonia digitata*, represent priority candidates for laboratory investigation, as their repeated independent documentation across unconnected populations provides stronger ethnopharmacological justification than single-study reports. Researchers looking for a clear starting point for extract preparation and bioassay screening should prioritise leaf and bark preparations from these species within the Fabaceae and Asteraceae families, as leaves were the most consistently used plant part and these two families dominated the documented dataset.

However, translating this traditional knowledge into evidence-based medicine is not straightforward. Several important challenges must be addressed before this botanical heritage can deliver its full potential. Firstly, there are significant environmental concerns. The heavy reliance on roots and bark documented in this review is destructive to plant populations when done without control, and several of the most-cited species already face pressures from overharvesting and habitat loss across sub-Saharan Africa. Any research programme targeting these plants must be paired with conservation action including cultivation trials, community-based propagation schemes, and the development of sustainable wild-harvesting protocols. This is to ensure that the plant resources themselves are not depleted in the process of scientific investigation. Secondly, the phytochemical composition of these plants is not fixed: it changes with soil type, rainfall, temperature, altitude, and the time of year at which the plant is collected. This means that results from one study cannot be reliably compared with another unless collection conditions are standardised. Researchers must therefore document and report collection metadata as a basic requirement, and future studies should incorporate multi-site and multi-season phytochemical profiling to understand and manage this natural variability.

Thirdly, and most critically, there are deep scientific data gaps that must be filled. The vast majority of the 556 species documented in this review are supported only by traditional use reports. Experimental evidence, predominantly in vitro cytotoxicity data, exists for only a small minority, and evidence from animal studies or human clinical trials is almost entirely absent. This is the central scientific challenge facing this field. Without systematic pharmacological validation, the ethnobotanical record, however rich, cannot support drug development claims. To begin filling this gap in a structured and efficient way, future studies should follow a clear research pathway. This can start with bioassay-guided fractionation of standardised extracts from the highest-priority species, moving to identification and isolation of the active compound or compounds responsible for the observed cytotoxicity, then determining their selectivity for cancer cells over normal cells, and finally conducting in vivo efficacy and toxicity studies in appropriate animal models before any consideration of human trials. Crude extract testing alone without progression toward compound identification is insufficient to build the scientific foundation that this field needs. The active concentrations of isolated compounds must be reported precisely, as these data are what make results meaningful and comparable across laboratories.

Fourthly, the geographic coverage of this review is uneven. Only 16 of sub-Saharan Africa’s 49 countries are represented in the included studies, and Central Africa is almost entirely absent. This does not mean that traditional cancer plant use is absent in these regions. Rather, it means research has not been done. Expanding primary ethnobotanical surveys to these underrepresented countries is an urgent priority, and such surveys should adopt standardised reporting frameworks to allow future data to be compared and pooled across studies. Alongside this, the indigenous knowledge systems that carry this botanical information must be actively protected. Traditional healers who hold this knowledge are ageing, and in many communities this knowledge is transmitted orally and is at risk of being lost entirely. Preserving traditional knowledge is therefore not only a cultural imperative but a scientific one. Without it, the leads for future drug discovery may be lost before they can even be studied.

In summary, sub-Saharan Africa’s traditional plant knowledge represents a genuinely valuable and largely untapped resource for anticancer drug discovery. Realising the value of these medicinal plants requires more than documenting plant use. It requires addressing environmental sustainability, standardising collection and preparation practices, filling the critical gaps in pharmacological and toxicological data, and giving researchers a clear and prioritised direction for where to begin. The Fabaceae, Asteraceae, Euphorbiaceae, Apocynaceae, and Lamiaceae families, and the cross-culturally documented species within them can be considered a starting point.

Future studies should adopt standardised ethnobotanical reporting frameworks, prioritise species with cross-cultural documentation, and work toward integrating validated plant-based therapies into evidence-based oncology practice in sub-Saharan Africa.

## Figures and Tables

**Table 1:** List of medicinal plants traditionally used as anti-cancer in Sub-Saharan Africa.

Family	Scientific Name	Common Name	Local Name	Plant Part Used	Mode of Preparation	Mode of Administration	Cancer Type	Claimant	Region	Reference
Acanthaceae	*Isoglossa ciliata* (Nees) Engl.	NA	Nandwasi	Leaves	Decoction or brunt	Oral, topical	Prostate, cervical, breast, colon, lung, GIT, skin, intestinal, uterine, oesophageal bone & bone	Elgon	Uganda	[23]
	*Asystasia gangetica* (L.) T.Anderson	Creeping foxglove	NA	Leaves	Decoction	Oral	Prostate	Ashanti	Ghana	[26]
	*Barleria cristata* L.	Philippine violet, bluebell barleria	NA	Leaves	Poultice		Stomach	Ashanti	Ghana	[26]
	*Dicliptera laxata* C.B.Clarke	NA	NA	Leaves, stem, bark	NA	Oral	Colon		Kenya	[12]
	*Eremomastax speciosa* (Hochst.) Cufod	NA	NA	Whole plant		Topical	Skin	NA	Eastern and Western Africa	[12]
	*Hypoestes aristata* Soland. ex Roem and Schult. var. *aristata*	Ribbon bush	Hlonyane, olukhulu (Zulu)	Leaves	NA	NA	Human drug-sensitive CCRF-CEM and multidrug-resistant CEM/ADR5000 leukaemia cells	Xhosa	South Africa	[15]
	*Justica betonica* L.	White shrimp plant, squirrel’s tail	NA	Leaves, stem, bark	NA	NA	Breast, colon, skin	NA	Kenya	[12]
	*Justicia extensa* T.Anderson	Steves’s leaves	NA	leaves	Decoction	Oral	Stomach	Ashanti	Ghana	[26]
	*Thunbergia alata* Bojer ex Sims	Black-eyed Susan	NA	leaves	tea	Oral	Stomach	Ashanti	Ghana	[26]
Alliaceae	*Allium sativum*	Garlic	Kachuum	Bulb	Decoction or burnt	Oral, topical	Cervical, colon, prostate, skin	Elgon	Uganda	[23]
	*Allium sativum* L.	Garlic	Aiyo	Fruits	Decoction	Oral	Lung and breast	Ewe	Togo	[11]
	*Tulbaghia violacea* Harv	Wild garlic, society garlic	Utswelane	Leaves, bulb	Decoctions	Orally	Oesophageal	Eastern cape	South Africa	[31]
Amaranthaceae	*Alternanthera sessilis* (L.) R.Br. ex DC	Dwarf copperleaf, Brazilian spinach		Leaves		Dressing	Stomach	Ashanti	Ghana	[26]
	*Amaranthus graecizans* L.	Mediterranean amaranth		Roots	Ground roots	NA	Brain	Ashanti	Ghana	[26]
	*Amaranthus hybridus* L.	Green amaranth, smooth pigweed		Leaves		Massage	Breast	Ashanti	Ghana	[26]
	*Amaranthus hybridus* L.	Green amaranth, smooth pigweed	Karatoutou	Root, leaves	Powdered	Topical, oral	Breast and cervix		Togo	[11]
	*Amarathus viridis* L.	Slender amaranth	NA	Leaves	Wound dressing, decoction	Oral, topical	prostate, breast, brain, stomach	Ashanti	Ghana	[26]
	*Beta vulgaris* L.	Beetroot	NA	bulb	Juice	Oral	Blood	Kampala	Uganda	[32]
	*Dysphania ambrosioides* (L.) Mosyakin & Clemants	Mexican tea, wormseed	NA	Leaves, bark	Ground leaves, decoction	Oral	Breast, brain, stomach, throat	Ashanti	Ghana	[26]
	*Aerva javanica* (Burm.f.) Juss. ex Schult	Kapok bush, dessert cotton	Tobia	Root	Powdered roots	Oral	All cancer	Ethiopia	Ethiopia	[20]
Amaryllidaceae	*Agapanthus africanus* (L.) Hoffmans	African lily	NA	Roots		Topical	Skin	NA	South Africa	[12]
	*Agapanthus africanus* (L.) Hoffmans	African lily, cape agapanthus	Mathunga	Root	Dried roots, powdered, infused in water	Oral	Uterine and breast	Eastern cape	South Africa	[31]
	*Allium cepa* L.	Common onion	NA	bulb	Excipient, grounded	Topical	Stomach, skin, liver	Ashanti	Ghana	[26]
	*Allium cepa* L.	Common onion	Kitungulu	bulbs	Mixed with *Mangifera indica,* *Aloe vera* & *Bidens pilosa,* boiled drunk (Concoction)	Oral	Stomach	Elgon	Uganda	[23]
	*Allium* *sativum* L.	Garlic	katungulusumu	Bulb	Chewed or cooked with food	Oral	Lung	Kampala	Uganda	[32]
	*Amaryllis belladonna* L.	Jersey lily, belladonna-lily	Maartblom, maartlelie (Afrikaans)	Leaves, flowers, bulbs	NA	Oral	Brain	Western Cape	South Africa	[12]
	*Boophone disticha* (L.f.) Herb.	Poison bulb, sore-eye flower	Perdespook (Afrikaans), motlatsisa (Sotho)	Leaves, flowers, bulbs	NA	NA	Brain	Western Cape	South Africa	[12]
	*Crinum abyssinicum* Hochst. ex A.Rich.	NA	Yegibb shinkurt	Leaves	NA	NA	All tumour	NA	Ethiopia	[20]
	*Crinum jagus* (J.Thomps.) Dandy	St. Christopher lily	NA	Leaves, flowers, bulbs	NA	NA	Brain	NA	Nigeria	[12]
	*Crinum stuhlmannii subsp. delagoense* (I. Verd.) Kwembeya & Nordal Syn. *Crinum delagoense* I. Verd.	Candy-striped crinum	NA	Leaves, flowers, bulbs	NA	NA	Brain	NA	Southern Africa	[12]
	*Tulbaghia violacea* Harv.	Wild garlic, sweet garlic	NA	Bulbs, leaves	NA	NA	Cervical	NA	Southern Africa	[12]
Anacardiaceae	*Anacardium occidentale* L.	cashew	NA	Leaves, bark	Decoction	Oral	Liver	Ashanti	Ghana	[26]
	*Cotinus coggygria* (Scop)	Smoke tree or smoke bush	NA	NA	NA	NA	Human drug-sensitive CCRF-CEM and multidrug-resistant CEM/ADR5000	NA	NA	[15]
	*Lannea acida* A.Rich.	NA	Kelo	Roots	Powdered	Topical	Chronic wound	Tem	Togo	[11]
	*Mangifera indica* L.	NA	NA	Bark, leaves	decoction, poultice, tea	Oral, topical	Lung, skin, prostate, lungs	Ashanti	Ghana	[26]
	*Mangifera indica* L.	Mango	mangoro	leaves	Decoction	Oral	NA	Mubi	Adamawa, Nigeria	[33]
	*Mangifera indica* L.	Mango	Mango	Leaves, stem back	Decoction	Oral	Breast, chronic wound	Kabyè	Togo	[11]
	*Mangifera* *indica* L.	Mango	Kiyembe	Leaves	Decoction or burnt	Oral, topical	Prostate, cervical, breast colon, lung, GIT, skin, intestinal, uterine, oesophageal bone & bone cancers	Elgon	Uganda	[23]
	*Pistacia vera* L.	Pistachio	NA	Oleum gum resin	NA	NA	Human drug-sensitive CCRF-CEM and multidrug-resistant CEM/ADR5000	NA	South Africa	[15]
	*Searsia pyroides* (Burch.) Moffett. Syn. *Rhus vulgaria* Meikle	Common wild currant	NA	Leaves, stem	NA	Topical	Stomach, skin, breast	NA	Kenya	[12]
	*Schinus molle* L.	Californian pepper tree	Rooioeoerbom (Afrikaans)	Leaves and resin	NA	NA	Human drug-sensitive CCRF-CEM and multidrug-resistant CEM/ADR5000	NA	South Africa	[15]
Anisophylleaceae	*Anisophyllea dichostyla* R.Br.	NA	NA	Roots	NA	NA	NA	NA	Central Africa, DRC	[12]
Annonaceae	*Annona muricata* L.	Soursop	NA	Fruits		Oral	Stomach	Ashanti	Ghana	[26]
	*Annona* *muricata* L.	Soursop	Kitafeeri, Obwolo	Root, leaves, fruits	Decoction	Oral	NA	Kampala	Uganda	[32]
	*Annona muricata* L.	Soursop	Agnigli	Leaves	Decoction	Oral	Breast, bone, lung	Ewè	Togo	[11]
	*Annona reticulata* L.	Wild sweetsop	NA	Leaves	Tea	Oral	Stomach, prostate	Ashanti	Ghana	[26]
	*Annona senegalensis* Pers.	Wild custard apple	NA	Fruits, leaves, bark	Juice, decoction	Oral	Stomach, throat, skin, breast	Ashanti	Ghana	[26]
	*Annona senegalensis* Pers.	Wild custard apple	Tchoutchoure	Root, leaves	Powdered	Topical	Bone, chronic wound	NA	Togo	[11]
	*Annonidium mannii* (Oliv.) Engl. & Diels	Jungle sop	NA	Fruits, leaves, and stem barks	NA	Oral	Leukaemia, breast, colon	NA	Central and West Africa	[12]
	*Enantia chlorantha* Oliv.	Awopa (Yoruba)	NA	Fruits, leaves, and stem barks	NA	Oral	Skin	NA	West Africa	[12]
	*Pachypodanthium staudtii* Engl. & Diels.	NA	NA	Fruits, leaves. Stem barks	Na	Oral	Leukaemia, breast, colon, Brain, liver	NA	East and West Africa	[12]
	*Uvaria chamea* P. Beauv.	Bush banana, finger root	NA	Fruits, leaves, and stem barks	NA	Oral	All types	NA	Nigeria	[12]
	*Xylopia aethiopica* (Dunal) A.Rich.	Grains of selim	Soussi	Fruits	Decoction, sauce	Oral, body bath	Bone, chronic wound, liver and breast	Kabyè	Togo	[11]
Apiaceae	*Daucus carota* L.	Carrots	NA	Root	Eat raw roots regularly; used with beetroot	Oral	Blood	Kampala, Wakiso	Uganda	[32]
	*Stegantaenia araliacea* Hochst	Carrot-tree,	Musvodzambudzi	Bark, roots	Decoction, infusion, tincture	Orally	Breast, skin, blood	Harare	Zimbabwe	[24]
	*Heteromorpha trifoliata* (Wendl.) Eckl. & Zeyh	Common parsley tree	Mhingano, imfenkulu	Leaves, bark, roots	Infusion and decoction	Orally	Skin, blood	Matebeleland	Zimbabwe	[24]
Apocynaceae	*Acokanthera oppositifolia* (Lam.) Codd	Bushman’s poison	inHlingunyembe (Zulu)	Leaves	Powdered snuff	Inhaling	Human drug-sensitive CCRF-CEM and multidrug-resistant CEM/ADR5000 leukaemia cells	NA	South Africa	[15]
	*Alafia multiflora* (Stapf) Stapf	Alafia	o-kum adada (Twi, Ghana)	Leaves and roots	Decoction	Oral	Breast, brain, skin, lung	Ashanti	Ghana	[26]
	*Alstonia boonei* De Wild.	God’s tree		Leaves, bark and roots	Ground exudates in lemon and decoction	Massage and oral	Breast, skin	Ahanti	Ghana	[26]
	*Calotropis procera* (Aiton) Dryand.	Apple of Sodom, Sodom apple	NA	Leaves	Decoction	Oral	Stomach, skin	Ashanti	Ghana	[26]
	*Carissa edulis* Vahl.	Climbing num-num		Roots, bark, leaves	NA	NA	NA	NA	Nigeria	[12]
	*Catharanthus roseus* (L.) G.Don	Madagascar periwinkle	Begraafplaasblom (Afrikaans)	Leaves and whole plant	Plant extract	orally	Breast, lung and uterine	Eastern cape	South Africa	[31]
	*Catharanthus* *roseus* (L.) G. Don	Madagascar periwinkle	Sekagya	Fruits	NA	NA	NA	NA	Uganda	[32]
	*Funtumia elastica* (Preuss) Stapf	Bush rubber tree, silkrubber	NA	Bark, leaves	decoction	Oral	Skin, throat, stomach, breast	Ashanti	Ghana	[26]
	*Gomphocarpus fruticosus* (L.) W.T Aiton	Milkweed, wild cotton	Lebegane (Sotho), Umsinga-Iwesalukazi (Zulu)	Roots, leaves, fruits and stems	NA	NA	breast MCF7, renal TK10 and melanoma UACC62	NA	South Africa	[12]
	*Gomphocarpus physocarpus* E. Mey.	Hairy balls, ballon wildcotton	Umsingalwesalukazi (Zulu)	Roots	NA	NA	breast MCF7, renal TK10 and melanoma UACC62	NA	South Africa	[12]
	*Holarrhena floribunda* (G.Don) T.Durand & Schinz	False rubber tree, conessi bark	NA	Leaves, bark	Decoction	Oral	Breast, brain, stomach	Ashanti	Ghana	[26]
	*Landolphia owariensis* P. Beauv.	White rubber vine, eta	NA	Climber	Alcoholic extract	Massage	Skin	Ashanti	Ghana	[26]
	*Picralima nitida* (Stapf) T. Durand &H. Durand	The akuamma, pile plant	NA	Fruits, bark	Decoction, tea	Oral, massage	Skin	Ashanti	Ghana	[26]
	*Pleiocarpa pycnantha* (K. Schum.) Stapf	NA	NA	Roots	Decoction	Oral	Breast	Ashanti	Ghana	[26]
	*Plumeria alba* L.	Caterpillar tree, pigeon wood	Ventupanier	Leaves, stem back	Decoction	Topical	Chronic wound	NA	Togo	[11]
	*Raphionacme hirsuta* (E. Mey.) R.A. Dyer	False gentian, khadi-root	Intesma (Xhosa), umathanjana (Zulu)	Leaves, flowers	NA	NA	NS	NA	Southern Africa	[12]
	*Rauvolfia vomitoria* Afzel.	Poison Devil’s pepper		Roots	Maceration, decoction	Oral	Genital, skin	Ashanti	Ghana	[26]
	*Rauvolfia* *vomitoria* Afzel	Poison devil’s-pepper	Kitondwani	Leaves, roots and stems	Decoction	Oral	Colon and cervical	Elgon	Uganda	[23]
	*Saba senegalensis* (A. DC) Pichon	Weda, madd	NA	Stem, climber	Tea	Oral	Stomach	Ashanti	Ghana	[26]
	*Strophanthus gratus* (Wall. & Hook.) Baill.	Woody lianas	NA	Roots	Decoction	Oral	Skin	Ashanti	Ghana	[26]
	*Tabernaemontana crassa* Benth.	Adam’s apple flower	NA	Leaves	Decoction	Oral	Lungs	Ashanti	Ghana	[26]
	*Tabernaemontana stapfina* Britten	Soccerball fruits	NA	Roots, leaves, stem barks	NA	NA	Breast	NA	Kenya	[12]
Araceae	*Colocasia* sp.	NA	NA	Leaves corn	Mixed with palm oil	Oral	Throat, prostate	Ashanti	Ghana	[26]
	*Colocasia esculenta* (L.) Schott	Taro	NA		Poultice and decoction	Topical, oral	Skin, breast	Ashanti	Ghana	[26]
	*Colocasia esculenta* (L.) *Schott*	Taro	Pankani	Leaves	Powdered	Oral	Breast	Kabyè	Togo	[11]
	*Xanthosoma sagittifolium* (L.) Schott	Coco yam, elephant ear	NA	Leaves	Exudate	Topical	Skin	Ashanti	Ghana	[26]
Araliaceae	*Centella asiatica* (L.) Urb.	Marsh pennywort or pepperwort	Icudwane (Zulu) or Varkoortjies (Afrikaans)	leaves	infusion	Oral	DU-145 prostate cancer cells, MDA-MB-231 and MCF-7 breast cancer cells	NA	South Africa	[34]
	*Cussonia bancoensis* Aubrev. & Pellegr.	Aky tree	NA	Bark	Decoction	Oral	Brain	Ashanti	Ghana	[26]
	*Cussonia paniculata* (Eckl. And Zeyh.)	Mountain cabbage tree	Umsengembuzi (Zulu)	Roots	NA	NA	breast MCF7, renal TK10 and melanoma UACC62	NA	South Africa	[12]
	*Panax ginseng* C.A. Meyer	Asian ginseng	Ginseng	Root	Decoction	Oral	Lung	NA	Togo	[11]
	*Polyscias fulva* (Hiern) Harms	Parasol tree	NA	Leaves	NA	NA	Leukaemia, breast, colon, brain, liver	NA	Sub-Sahara Africa	[12]
Arecaceae	*Butia capitata (Mart.)* *Becc.*	Jelly palm	Foda kokolo	Roots, fruits	Decoction	Oral	Breast	Kabyè	Togo	[11]
	*Cocos nucifera* L.	Coconut tree	NA	Roots	Decoction	Oral	Stomach, lungs	Ashanti	Ghana	[26]
	*Elaeis guineensis* Jacq.	African oil palm	NA	Fruits	Extract	Topical	Skin, genital	Ashanti	Ghana	[26]
	*Elaeis guineensis* f. androgyna A. Chev.	African oil palm	Pawou	Root	Decoction	Oral, body bath	Chronic wound	Kabyè	Togo	[11]
	*Eremospatha macrocarpa* H. Wendl.	Small rattan palm	NA	Bark	Tea	Oral	Skin	Ashanti	Ghana	[26]
Asclepiadaceae	*Calotropis procera* (Aiton) R.Br.	NA	Kpakpadjoe	Root, leaves	Decoction	Oral, topical	Breast and chronic wound	Kabyè	Togo	[11]
	*Leptadenia hastata* (Pers.) Decne	Anvara	Yadiya	Leaves	Decoction	Oral	NA	Mubi	Adamawa, Nigeria	[33]
	*Periploca nigrescens* Afzel.	NA	NA	leaves	Tea	Oral	Skin, prostate, throat, breast	Ashanti	Ghana	[26]
Asparagaceae	*Asparagus africanus* Lam.	Wild asparagus, asparagus fern	Rukato	Roots and leaves	Decoction, infusion	NA	Skin, prostate	Mashnaland central	Zimbabwe	[24]
	*Asparagus africanus*	Wild asparagus	Yeset kest	Roots	NA	NA	Uterine, breast	NA	Ethiopia	[20]
	*Fusifilum depressum* (Baker) U. Mull. -Doblies, J.S.Tang & D.Mull.-Doblies	NA	NA	Leaves, bulbs	NA	NA	NS	NA	Southern Africa	[12]
Asphodelaceae	*Aloe ferox* Mill	Bitter aloe, red aloe	iNhlaba (Zulu), iKhala (Xhosa)	Roots, leaves	NA	NA	Skin	NA	Southern Africa	[12]
	*Aloe vera*	True aloe	Kigadi	Leaves	Decoction Burnt & applied on infected skin	Oral, topical	Prostate, cervical, breast colon, lung, skin intestinal uterine, oesophageal bone & bone	Elgon	Uganda	[23]
	*Aloe volkensii* Engl.	NA	NA	Leaves, stem barks	NA	NA	Colon, oesophageal, prostate	NA	Kenya	[12]
	*Bulbinella floribunda* (Thunb.) T. Durand &Schinz.	Cat’s tail, yellow cat-tail	Chidzinganyoka	Leaves	NA	NA	Breast, prostate, colon	Bulawayo	Zimbabwe	[24]
Asteraceae	*Acanthospermum* *hispidum* DC.	Goat’s head, hispid starburr	Lan gbanisoe	Leaves	Decoction	Oral	Bone and skin	Kabyè	Togo	[11]
	*Ageratum conyzoides* (L.) L.	Billygoat weed	NA	Leaves, roots, bark, whole plants	Decoction, paste, crushed fresh leaves for juice	Oral and topical	Skin, cervical, throat, breast, lung	Ashanti	Ghana	[26]
	*Artemisia afra* Jacq. ex Willd.	Wild wormwood, African wormwood	Umhlonyane (isiXhosa)	Leaves, stem	NA	NA	NA	NA	South Africa	[12]
	*Artemisia* *annua* L.	Sweet sagewort, annual wormwood	NA	Leaves	Infusion with rock salt	Oral	NA	NA	Uganda	[32]
	*Artemisia armeniaca* Lam.	NA	NA	Leaves	NA	NA	NA	NA	South Africa	[12]
	*Artemisia indica* Willd.	Indian wormwood	NA	Leaves	NA	NA	NA	NA	South Africa	[12]
	*Aspilia africana* (Pers.) C.D.Adams	Wild sunflower, haemorrhage plant	NA	leaves	Decoction	Oral	Lungs	Ashanti	Ghana	[26]
	*Athrixia elata* Sond.	Daisy tea bush	NA	Leaves, seeds	NA	NA	breast MCF7, renal TK10 and melanoma UACC62	NA	South Africa	[12]
	*Bacchariodes lasiopus* (O. Hoffm.) H.Rob.	NA	NA	Leaves, stem bark	NA	NA	Colon	NA	Kenya	[12]
	*Bidens pilosa* L.	NA	NA	Whole plant	Tea	Oral	Breast	Ashanti	Ghana	[26]
	*Bidens pilosa* L.	Kyikhamama (Tabululu)	NA	Leaves, stem and roots	Boiled or burnt	Oral, topical	Prostate, cervical, breast colon, lung, GIT, skin, intestinal uterine, oesophageal bone & bone	Elgon	Uganda	[23]
	*Bidens pilosa* L.	Blackjack, Cobbler’s peg	Umesis or isikhathula (Zulu)	Leaves and stems	Infusion	Oral	DU-145 prostate cancer cells, MDA-MB-231 and MCF-7 breast cancer cells	NA	South Africa	[34]
	*Chromolaena odorata* (L.) R.M.King & H.Rob	Siam weed, devil weed, Christmas bush	NA	Leaves	Maceration, poultice, decoction, ground leaves in mixture	Oral, topical	Skin	Ashanti	Ghana	[26]
	*Cnicus benedictus* L.	Bitter thistle	Makdissel (Afrikaans)	leaves	decoction	Oral	DU-145 prostate cancer cells, MDA-MB-231 and MCF-7 breast cancer cells	NA	South Africa.	[34]
	*Conyza sumatrensis* (Retz.) E. Walker	White horseweed, fleabane	Namagoye	Leaves	Mixed *Dicrocephala integrifolia,* boiled) or burnt	Oral, topical	Prostate, cervical, breast, colon, lung, GIT, skin intestinal, uterine, oesophageal bone & bone	Elgon	Uganda	[23]
	*Dicoma anomala*	Stomach or fever bush	Umuna (Zulu), inyongana (Swazi, Xhosa)	Leaves and roots	Decoction	Oral	NA	NA	South Africa	[14]
	*Dicoma capensis* Less.	Fever bush	Koorsbossie (Afrikaans)	Leaves and twigs	Infusion	Oral	DU-145 prostate cancer cells, MDA-MB-231 and MCF-7 breast cancer cells	NA	South Africa	[34]
	*Dicrocephala integrifolia* (L.f) Kuntze	NA	Lunyabakana	Leaves	Mixed with *Vernonia adonsis* and *Mangifera indica*, boiled or burnt	Oral, topical	Prostate, cervical, breast, colon, lung, GIT, skin, intestinal uterine, oesophageal bone & bone.	Elgon	Uganda	[23]
	*Elephantopus mollis* Kunth.	Tobacco weed, soft, elephant’s foot	NA	Leaves	NA	NA	Brain	NA	Cameroon	[12]
	*Elytropappus rhinocerotis* (L.f.) Less.	Rhinoceros bush or	Renosterbos (Afrikaans)	Whole plants	Infusion of young branches in wine or brandy	Oral	Stomach	Eastern cape	South Africa	[31]
	*Emilia sonchifolia* (L.) DC. Ex DC.	Lilac tassel flower	NA	Whole plant	Paste	Oral/topical	Stomach, skin	Ashanti	Ghana	[26]
	*Ethulia conyzoides* L.f.	Blue weed	NA	Whole plant	Squeezed juice	Oral	Lungs, skin, breast	Ashanti	Ghana	[26]
	*Galinsonga parviflora* Cav.	Gallant soldier, quickweed, potato weed	NA	Leaves	NA	NA	Colon	NA	Kenya	[12]
	*Microglossa pyrifolia* (Lam.) Kuntze	NA	NA	Leaves, stem	NA	NA	NA	NA	Kenya	[12]
	*Piptocarpha riedelii* (Sch.Bip) Baker	Ashdaisy	NA	Leaves	Decoction	Oral	Prostate, lungs, liver	Ashanti	Ghana	[26]
	*Senecio glanduloso-pilosus* Volkens & Muschl.	NA	NA	Leaves	NA	NA	NA	Free State, Limpopo, Mpumalanga, Northwest	Southern Africa	[12]
	*Senecio incomptus* DC.	NA	NA	Leaves	NA	NA	NA	Eastern Cape	Southern Africa	[12]
	*Solanecio manii* (Hook.f.) C. Jeffery	Gynura manni	Mukono (Rukiga), Kiralankuba (Luganda)	Leaves, stem barks	NA	NA	Skin, breast, colon	NA	Kenya	[12]
	*Solanecio nandensis* (S. Moore) C. Jeffrey	NA	NA	Leaves, stem barks	NA	NA	NS	NA	Kenya	[12]
	*Sonchus oleraceus* L.	Common sowthistle	NA	Leaves, flowers	NA	NA	NA	NA	South Africa	[12]
	*Tithonia diversifolia* (Hemsl. A. Gray)	Mexican sunflower	NA	Leaves	NA	NA	breast MCF7, renal TK10 and melanoma UACC62	NA	South Africa	[12]
	*Vernonia adoensis* (Sch. Bip. ex Walp.) H.Rob	NA	Kisola	Leaves	Mixed with *Kigelia africana,* *Ananas sativa* (Retz) Walker boiled, burnt	Oral, topical	Prostate, cervical, breast, colon, lung, GIT, skin, intestinal, uterine, oesophageal bone & bone	Elgon	Uganda	[23]
	*Xanthium strumarium* L.	Rough cocklebur	NA	Stems	NA	NA	breast MCF7, renal TK10 and melanoma UACC62	NA	South Africa	[12]
	*Zinnia peruviana* (L.) L.	*Peruvian zinnia*		NA	Leaves, flowers	NA	breast MCF7, renal TK10 and melanoma UACC62	NA	South Africa	[12]
Balanophoraceae	*Sarcophyte sanguinea* Sparrm. subsp. *sanguinea*	NA	Wolwekos (Afrikaans), umavmbuka (isiZulu)	Whole plants	Decoction of the whole plant	Oral	NA	Eastern Cape	South Africa	[31]
Bignoniaceae	*Kigelia africana* (Lam.) Benth.	Sausage tree	Abiliou/Tem, Lèmirè/Kabyè	Leaves, stem back	Maceration	Body bath	Breast	Tem, Kabyè	Togo	[11]
	*Kigelia africana* (Lam.) Benth.	Sausage tree	Gufungo/Kifungo	Leaves, roots and stems	Mixed with *Hydnora abyssinica* A and *Hydnora Africana,* boiled or burnt	Oral/topical	Breast and prostate	Elgon	Uganda	[23]
	*Kigelia africana* (Lam.) Benth.	Sausage tree	NA	Leaves, back	Ground leaves, decoction	Topical, oral	Skin, prostate	Ashanti	Ghana	[26]
	*Kigelia* *africana* (Lam.) Benth.	Sausage tree	Edodoi, yago,	Roots, bulbs	Crushed in water to make concoction, 1–2 tablespoon of juice taken twice daily	Oral	NA	Tororo/ Mbale	Uganda	[32]
	*Kigelia africana* (Lam.) Benth.	Sausage tree	Mubveve	Fruits, leaves, bark	NA	Oral	Blood, skin	Manicaland	Zimbabwe	[24]
	*Markhamia* *lutea* (Benth.) K. Schum.	Nile tulip, siala tree	Sambya, Lusambya, Lusoola	Fruits	NA	NA	NS	NA	Uganda	[32]
	*Markhamia lutea (Benth.)* K. Schum.	NA	NA	Leaves, Stem barks	NA	NA	Colon	NA	Kenya	[12]
	*Markhamia tomentosa* (Benth.) K. Schum. ex Engl	Siala	Akoko (Yoruba) Abakansi (Igbo)	Leaves, flowers, stem backs	NA	NA	Cervical	NA	West Africa	[12]
	*Newbouldia laevis* (P. Beauv.) Seem.	Boundary tree	NA	Bark, leaves, root	Ground leaves, decoction, tea	Topical, oral	Prostate, breast, ovarian	Ashanti	Ghana	[26]
	*Spathodea campanulata* P. Beauv.	African tulip tree	NA	Bark, leaves	Decoction	Oral	Stomach, skin, throat	Ashanti	Ghana	[26]
	*Tecoma stans* (L.) Juss. Ex Kunth	Yellow elder, ginger Thomas	NA	Leaves	Tea	Oral	Skin, breast	Ashanti	Ghana	[26]
Bombacaceae	*Adansonia digitata* L.	African baobab	Tèlou	Stem back, fruits	Decoction, powder, sauce	Topical, oral	Chronic wound, breast	Kabyè	Togo	[11]
Boraginaceae	*Cordia millenii* Baker	Cordia wood, drum tree	Kyeneboa	Bark	Inhalation	Nasal	Lungs	Ashanti	Ghana	[26]
	*Cordia myxa* L.	Assyrian plum	NA	Leaves	Decoction	Oral	Stomach, brain, breast	Ashanti	Ghana	[26]
	*Cordia vignei* Hutch. & Dalziel	NA	NA	Bark	Decoction	Oral	Prostate	Ashanti	Ghana	[26]
	*Heliotropium indicum* L.	Indian heliotrope, Indian turnsole	NA	Leaves	Tincture, decoction, maceration, ground leaves	Oral and topical	Skin, breast, prostate, stomach, throat	Ashanti	Ghana	[26]
Brassicaceae	*Sinapis alba* L.	NA	NA	Seeds	NA	NA	NA	Ashanti	Ghana	[26]
Bromeliaceae	*Ananas comosus* (L.) Merr.	Pineapple	NA	fruits	Juice, decoction, extract	Oral	Lungs	Ashanti	Ghana	[26]
	*Ananas sativus* (Retz) Walker		Nadanga	Leaves, roots and stems	Boiled and drunk (decoction) *Mixed with Erythrina abyssinica* Lam., and *Mormordica foetida*, boiled & drunk (concoction)	Oral	Oesophageal	Elgon	Uganda	[23]
Burseraceae	*Canarium* *schweinfurthii* Engl.	Bush candle, African olive	Mwafu, Mubafu	Fruits	Eaten raw	Oral	NA	NA	Uganda	[32]
	*Commiphora africana* (A.Rich.) Endl	African myrrh	Dashi	Leaves	Decoction	Orally	NA	Mubi	Adamawa, Nigeria	[33]
	*Commiphora africana* (A.Rich) Endl	African myrrh		Whole plant	Smoking	Inhalation	lungs	Ashanti	Ghana	[26]
	*Boswellia dalzielii* Hutch.	Frankincense tree	Harrabi	Bark	Decoction	Orally	NA	Mubi	Adamawa, Nigeria	[33]
Cactaceae	*Opuntia* species	Prickly pear cactus	NA	Leaves	Juice squeezed from leaves	Oral	Prostate, stomach, colon and rectum	Kampala	Uganda	[32]
Calophyllaceae	*Mammea africana* Sabine	NA	NA	Bark resin, stem bark, roots	taped into shea butter, boil/ ground bark for dressing, decoction	Oral, topical	Cervical, breast, skin, throat	Ashanti	Ghana	[26]
Cannabaceae	*Cannabis sativa* L.	Marijuana	Umya	Leaves	Leaves are crushed	Oral	Skin	Eastern Cape	South Africa	[31]
	*Cannabis sativa* L.	Marijuana	Mbanje	Leaves, whole plant	Infusion, decoction, smoked, chewed or put in tea	Oral	All cancers	Harare	Zimbabwe	[24]
	*Celtis africana* Burm.f.	White stinkwood	umVumvu	Bark and leaves	Infusion in milk	Oral	NA	Eastern Cape	South Africa	[31]
Caricaceae	*Carica papaya* L.	pawpaw	NA	Leaves	NA	NA	Cervical, colon, breast	NA	Kenya, Guninea, Nigeria, Cameroon, Ghana	[12]
	*Carica papaya* L.		NA	Leaves, seeds, roots	Tea	Oral	Stomach, skin, breast, prostate	Ashanti	Ghana	[26]
	*Carica papaya* L.		Sombre	Roots	Powdered	Oral	Lung	Kabye	Togo	[11]
	*Euclea* *natalensis* A.DC.		NA	Bulb	Decoction	Oral	Prostate	Mukono/ Buikwe	Uganda	[32]
Casuarinaceae	*Casuarina equisetifolia* L.	NA	NA	Bark, kennel	Nasal drops	Nasal	Brain	Ashanti	Ghana	[26]
Caesalpiniaceae	*Afzelia africana* Sm. ex Pers.	African mahogany	Wèrè	Stem back, roots	Powdered	Topical	Chronic wound, skin, bone	Kabyè	Togo	[11]
	*Caesalpinia pulcherrima* (L.) Sw.	Peacock flower	Wayi	Leaves	Decoction	Oral	Prostate	Kabyè	Togo	[11]
	*Daniellia oliveri* (Rolfe) Hutch. & Dalziel	West African copal tree	Tchèlè	Leaves	Sauce	Oral	lung	Tem	Togo	[11]
	*Piliostigma thonningii* (Schumach.) MilneRedh	Camel’s foot tree, monkey bread	Eclo/Ewe, Babakou/Kabyè	Leaves, root	Powdered, sauce	Oral, topical	Skin, lung, chronic wound, prostate, breast	Ewe, Kabyè	Togo	[11]
	*Tamarindus indica* L.		Nidie	Fruits	Powdered	Topical	Breast	Kabye	Togo	[11]
Celastraceae	*Catha edulis* (Vahl.) Endl.	Bushman’s tea	Iqgwaka (Xhosa)	Leaves, fruits			breast MCF7, renal TK10 and melanoma UACC62		South Africa	[12]
	*Gymnosporia* *senegalensis* (Lam.) Loes.	NA	Tchakatchaka	Leaves	Decoction	Oral and topical	Chronic wound	Kabye	Togo	[11]
	*Gymnosporia tenuispina* (Sond.) Szyszyl.	Bell spike-thorn	Klokkies-pendoring (Afrikaans)	Leaves, fruits	NA	NA	breast MCF7, renal TK10 and melanoma UACC62	NA	South Africa	[12]
	*Loeseneriella africana* (Willd.)	African paddle-pod	NA	Leaves, stem barks	NA	NA	Colon, breast	NA	Kenya	[12]
	*Maytenus senegalensis* (Lam.) Exell. (*syn. Gymnosporia senegalensis* (Lam.) Loes.)	Red spike thorn	NA	Roots	Decoction	Oral	Prostate	Ashanti	Ghana	[26]
	*Maytenus senegalensis* (Lam.) Exell	Red spike thorn, confetti tree	Kikonje	Leaves, roots and stems	Decoction	Oral	Cervical	Elgon	Uganda	[23]
Chrysobalanaceae	*Parinari curatellifolia* Planch. ex Benth.		Milioumiliou	Leaves, roots and root back	Sauce, decoction, powder	Oral,	Lung, chronic wound, breast	Tem	Togo	[11]
Clusiaceae	*Allanblackia monticola* Staner L.C.			Leaves, seeds			Leukaemia		Cameroon	[12]
	*Garcinia buchananii* Baker	Granite garcinia, granite mangosteen	Kikameli	Leaves, stems	Decoction	Oral	Prostate, cervical, breast colon, lung, GIT, skin intestinal uterine, oesophageal bone & bone cancers	Elgon	Uganda	[23]
	*Garcinia buchananii* Baker	NA	Kikameli	Leaves and stem	Decoction, mixed with *Rinus communis* and *Dioscaena fragran*	Oral	Prostate, cervical, breast colon, lung, GIT, skin intestinal uterine, oesophageal bone & bone	NA	Uganda	[23]
	*Garcinia kola* Heckel	Bitter kola	NA	Barks, roots, leaves	Decction, ground	Oral, topical	Skin, breast	Ashanti	Ghana	[26]
	*Garcinia punctata* Oliv.	NA	NA	Leaves, flowers	NA	NA	Colon	NA	NA	[12]
	*Hypericum lanceolatum* Lam	Wort	NA	Leaves, flowers	NA	NA	Colon	NA	NA	[12]
Cochlospermaceae	*Cochlospermum* *planchonii Hook. F.*	NA	Tekalichoua	Roots	Powdered	Oral	Breast	Kabye	Togo	[11]
Combretaceae	*Guiera senegalensis* J.F. Gmel	Sabara	Sabara	leaves	Decoction	Oral	NA	Mubi	Adamawa, Nigeria	[33]
	*Anogeissus leiocarpa* (DC.) Guill. & Perr	African birch	Kane, akuta	Roots	Tea	Oral	Skin	Ashanti	Ghana	[26]
	*Combretum collinum* Fresen.	Bicoloured bushwillow	Fokizao	Roots	Sauce	Oral	Lung	Tem	Togo	[11]
	*Combretum molle* R.Br. ex G.Don	Velvet bushwillow	NA	Leaves	Tea	Oral	Breast	Ashanti	Ghana	[26]
	*Combretum molle* G.Don	NA	Kimwanyimwayi	Leaves	Decoction	Oral, topical	Skin, prostate, cervical	Elgon	Uganda	[23]
	*Combretum kraussi Hochst.*	Forest bushwillo	Ulandile (Xhosa)	Leaves	NA	NA	Human drug-sensitive CCRF-CEM and multidrug-resistant CEM/ADR5000	NA	South Africa	[15]
	*Combretum platypterum* (Welw.) Hutch. & Dalziel	Red wings, dwarf red combretum	NA	Bark	Decoction	Oral	Skin, lungs	Ashanti	Ghana	[26]
	*Combretum racemosum* P.Beauv	English Christmas rose, false bougainvillea	o-hwiremo	Leaves	Decoction	Oral	Throat, breast	Ashanti	Ghana	[26]
	*Pteleopsis suberosa* Engl. & Diels	NA	Sisinon	Leaves	powdered	Oral	Chronic wound, lung	Tem	Togo	[11]
	*Terminalia catappa* L.	Indian almond, false kamani	NA	Leaves, back, fruits root, bark	Decoction, tea, nut for oil	Topical, oral	Stomach, skin, breast	Ashanti	Ghana	[26]
	*Terminalia ivorensis* A.Chev	Ivory coast almond, black afara	NA	Leaves, root bark	Poultice, decoction	Topical, oral	Skin, lungs	Ashanti	Ghana	[26]
	*Terminalia superba* Engl. & Diels	African limba wood	Ofram	Leaves, stem bark, root bark	Decoction, ground leaves	Oral	Stomach, lungs, prostate, skin	Ashanti	Ghana	[26]
Compositae	*Vernonia amygdalina*	Bitter leaves	grawa	leaves	NA	NA	All cancer		Ethiopia	[20]
	*Vernonia hymenolepis* A. Rich	Sweet bitter leaf	grawa	Leaves, stem	NA	NA	All cancer		Ethiopia	[20]
	*Cnestis ferruginea* Vahl ex DC.	NA	NA	Roots	Ground into powder for dressing	Topical	NA	Ashanti	Ghana	[26]
Convolvulaceae	*Ipomoea cairica* (L.)	Coast morning glory, mile-a-minute vine	NA	Leaves, flowers	NA	NA	Breast, cervical, skin	NA	Kenya	[12]
Cornaceae	*Curtisia dentata* (Burm.f.) C.A.Sm.	Assegai	UmLahleni, UmGxina	Bark, leaves	Dried bark and leaves are pulverised and infused in milk	Oral	Oesophageal	Eastern Cape	South Africa	[31]
Crassulaceae	*Bryophyllum pinnatum* (Lam.) Oken	NA	NA	Leaves, roots	Squeezed leaves, decoction	Topical, oral	Skin, stomach	Ashanti	Ghana	[26]
	*Cotyledon orbiculata* spp. *oblonga* (L.)	Pig’s ears, round-leafed navel-wort	Varkoor, kouterie (Afrikaans)	Roots	NA	NA	breast MCF7, renal TK10 and melanoma UACC62	NA	South Africa	[12]
	*Kalanchoe integra* (Medik.) Kuntze	NA	NA	Bark	Decoction	Oral	Breast	Ashanti	Ghana	[26]
	*Kalanchoe petitiana* A. Rich	NA	Indahula	Leaves	Decoction	Oral	Breast, skin	NA	Ethiopia	[20]
	*Kalanchoe paniculata* Harv.		Hasieoor (Afrikaans), indabulaluvalo (Zulu)	Roots	NA	NA	breast MCF7, renal TK10 and melanoma UACC62	NA	South Africa	[12]
Cucurbitaceae	*Cucurbita maxima* Duchesne	NA	NA	Leaves	Decoction	Oral	Lungs, head	Ashanti	Ghana	[26]
	*Cucumis prophetarum/ficifolius*	Wild cucumber	Yemdir embuay	roots	Roots are pulverised and made as pastes or ointments	Topical	Skin	NA	Ethiopia	[20]
	*Momordica angustisepala* Harms	NA	NA	Leaves	Tincture	Topical	Skin	Ashanti	Ghana	[26]
	*Momordica charantia* L.	Bitter melon	NA	Fruits, root	dried ground fruits for dressing wound, decoction	Topical, oral	cervical, breast, skin, stomach	Ashanti	Ghana	[26]
	*Momordica charantia* L.	Bitter melon	Katchala	Leaves	Powdered	Oral	Brain	NA	Togo	[11]
	*Momordica foetida* Schumach	Wild cucumber	NA	Leaves, stem barks	NA	NA	Breast, cervical	NA	Kenya	[12]
	*Lagenaria siceraria* (Mol.) Standl	Calabash, bottle gourd	Qui/kil	Leaves	Crushed or squeezed for the juice	Topical	Cancerous sore	NA	Ethiopia	[20]
Cupressaceae	*Juniperus procera* Hochst. ex Endl.	African juniper, Kenya-cedar	NA	Leaves, stem barks	NA	NA	Breast, throat, oral	NA	Kenya	[12]
Curtisiaceae	*Curtisia dentata* (Burm.f.)	Assegai tree, Cape lancewood	umLahleni (Zulu, Xhosa)	Leaves, stem barks	NA	NA	All types	NA	South Africa	[12]
Cyperaceae	*Cyperus rotundus* L.	NA	NA	Bulb, nut	NA	Oral	Stomach, lungs	Ashanti	Ghana	[26]
Dioscoreaceae,	*Dioscorea alata* L.	Purple yam, winged yam, water yam	NA	Tubers	Grounded	Topical	Skin	Ashanti	Ghana	[26]
	*Dioscorea bulbifera* L.	Bitter yam, parsnip yam, air-potato	Liakunyu	Leaves and tubers	Decoction, mixed with *Dioscaena fragrans* (L.) Ker-Gawl and *Cajanus Cajan*	Oral	Cervical, breast and prostate	Elgon	Uganda	[23]
	*Dioscorea bulbifera* L.	Bitter yam, parsnip yam, air-potato	NA	Tubers	Decoction, paste	Oral, topical	Skin, prostate, lungs	Ashanti	Ghana	[26]
	*Dioscorea cayennensis* Lam	Lagos yam	Nkani	Roots	Plaster with lemon, decoction	Oral	Brain	Ashanti	Ghana	[26]
	*Dioscorea cayennensis* subsp. Rotundata (Poir.) J. Miège	Yellow yam, Guinea yam	Nkani	Leaves, roots	Boiled extract, cooked roots	Oral	Breast, skin, prostate, liver	Ashanti	Ghana	[26]
	*Dioscorea dumetorum* (Kunth) Pax	Bitter yam, cluster yam	NA	Leaves	Decoction	Oral	Breast	Ashanti	Ghana	[26]
Dracaenaceae	*Dracaena fragrans* (L.) Ker Gawl.	Corn plant, striped dracaena	Linzi	Leaves	Decoction	Oral	Cervical, colon	Elgon	Uganda	[23]
Dryopteridaceae	*Cyrtomium falcatum* (L.f.) C.Presl	House holly-fern		Leaves, roots	NA	NA	Prostate		N/S	[12]
Ebenaceae	*Diospyros whyteana* (L.C)	Bladdernut, blackbark	umKahze (Zulu)	Leaves, flowers	NA	NA	breast MCF7, renal TK10 and melanoma UACC62	All provinces	South Africa	[12]
Euphorbiaceae	*Acalypha ciliata* Forssk.	NA	Mfofoa, Kpando	Leaves	Decoction	Oral	Breast	Ashanti	Ghana	[26]
	*Acalypha wilkesiana* (Muell.Arg.) Fosberg.	Copperleaf, Jacob’s coat	NA	Leaves, stem	NA	NA	Breast	NA	West Africa	[12]
	*Alchornea cordifolia* (Schumach. & Thonn.) Mull.Arg.	Christmas bush	NA	Leaves, bark	Decoction		Brain, stomach	Ashanti	Ghana	[26]
	*Astraea lobata* (L.) Klotzsch (syn. *Croton lobatus* L.)	NA	NA	Leaves	Decoction	Oral, topical	Skin	Ashanti	Ghana	[26]
	*Bridelia ferruginea* Benth.	NA	Kolou	Root	Powdered	Topical	Chronic wound	Tem	Togo	[11]
	*Bridelia micrantha* (Hochst.) Baillon	Coastal golden-leaf	NA	Leaves, stem barks	NA	NA	Cervical, skin, breast, colon	NA	Kenya	[12]
	*Croton hirtus* L’Her	Hairy croton	NA	Leaves	Decoction	Oral, topical	prostate, skin, breast, throat	Ashanti	Ghana	[26]
	*Croton macrostachyus* Hochst. ex Delile	NA	NA	Leaves, stem barks	NA	NA	Colon, skin	NA	Kenya	[12]
	*Elaeophorbia drupifera* (Thonn.) Stapf.	NA	NA	Leaves, stem barks	NA	NA	Leukaemia, breast, colon, Brain, liver	NA	West Africa	[12]
	*Euphorbia abyssinica* J.F.Gmel.	Desert candle	Kulkuwal	Roots, Latex	Pulverised roots	Topical	Skin	NA	Ethiopia	[20]
	*Euphorbia heterophylla* L.			Leaves	Decoction	Topical, oral	Throat, prostate, skin, breast	Ashanti	Ghana	[26]
	*Euphorbia hyssopifolia* L.	Hyssop leaf sandmat	NA	Leaves	Decoction	Oral, topical	Skin, prostate, breast, throat	Ashanti	Ghana	[26]
	*Euphorbia hirta* L.	Asthma-plant	NA	Leaves	Decoction	Oral/topical	Stomach, prostate, skin, breast, throat	Ashanti	Ghana	[26]
	*Euphorbia ingens* E.Mey. ex Boiss	Common tree Euphorbia	Nkonde	Latex	Latex	Topical	Skin	Eastern Cape	South Africa	[31]
	*Euphorbia tirucalli* L.	Pencil cactus, rubber-hedge euphorbia	kinchib	Roots, latex	Powdered roots	NA	All cancer	NA	Ethiopia	[20]
	*Jatropha curcas* L.	Physic nut, bubble bush, purging nut	NA	Leaves, bark, rotos	Leaves mix with oil, ground back to plaster, decoction	Oral	Skin, breast, prostate, stomach, brain	Ashanti	Ghana	[26]
	*Jatropha curcas* L.	Physic nut, bubble bush, purging nut	NA	Seeds	Paste made with seed powder and honey	Oral, topical	All cancer	NA	Ethiopia	[20]
	*Jatropha gossypiifolia* L.	Bellyache bush, cotton-leaf physic nut	NA	Leaves, roots, bark	NA	NA	Stomach (liver)	Ashanti	Ghana	[26]
	*Margaritaria discoidea* (Baill.) Müll.Arg.	Bushveld peacock-berry	Gulumati/Kilumati	Leaves, stem bark	Decoction	Oral, topical	Prostate, colon, cervical, breast	Elgon	Uganda	[23]
	*Mallotus oppositifolius* (Geiseler) Mll. Arg	Mallotus		Leaves, roots	Decoction, ground	Topical, oral	Genital, skin, prostate, breast, throat	Ashanti	Ghana	[26]
	*Manihot esculenta* Crantz	Cassava	NA	Tuber, roots, leaves	Grated and squeezed liquid, extract with salt	Oral, topical	Cervical, skin, genital	Ashanti	Ghana	[26]
	*Phyllanthus muellerianus* (Kuntze) Exell.	NA	Nbirè Nbirè	Root	Decoction	Oral	Bone	Tem	Togo	[11]
	*Ricinus communis* L.	NA	Mukakale	Leaves, roots, stem	Decoction, mixed with *Entada abyssinica* stued & *Mangfera indica*	Oral	Uterus	Elgon	Uganda	[23]
	*Ricinus communis* L.	Castor oil plant, castor bean	Mupfuta	Rots, leaves, bark	Decoction	Orally	Blood, colon	Mashonaland East	Zimbabwe	[24]
	*Tragia brevipes* Pax			Leaves, stem barks			Breast, leukaemia		Kenya	[12]
Fabaceae	*Abrus precatorius* L.	Coral bean or crabs’ eye	Umkhokha (Zulu)	Leaves	decoction	Oral	NA		South Africa	[12]
	*Abrus precatorius*	Coral bean	Adodobia fadi	Leaves	Powdered	Oral	Lung	Tem	Togo	[11]
	*Aeschynomene abyssinica* (A.Rich.)	Joint vetches		Leaves, fruits	NA	NA	NA	NA	Kenya	[12]
	*Albizia adianthifolia* (Schum.) W. Wight.	Flat-crown albizia	NA	Leaves	NA	NA	Leukaemia, breast, brain, colon	NA	Sub- Saharan Africa	[12]
	*Albizia coriaria* (Welw.) ex Oliver	NA	Kiluku	Root bark	Decoction	Oral	Prostate, cervical, breast colon, lung, GIT, skin intestinal uterine, oesophageal bone & bone	Elgon	Uganda	[23]
	*Albizia* *coriaria* (Welw.) ex Oliver	NA	Omugavu, Musiita, Kiluku	Bark	Decoction or ointment	Oral, topical	NA	NA	Uganda	[32]
	*Albizia grandibracteata* Taub.	Silk plants	NA	Leaves	NA	NA	Breast, cervical	NA	Uganda	[12]
	*Albizia gummifera* (J. F. Gmel.) C. A. Sm.	Peacock flower	NA	Leaves, fruits	NA	NA	Throat, skin	NA	Kenya	[12]
	*Amphimas pterocarpoides* Harms	Tropical timbers	NA	Leaves	Decoction	Oral	Head	Ashanti	Ghana	[26]
	*Andira inermis* (W. Wright) Kunth ex DC.	NA	NA	Leaves	NA	NA	NA	NA	Nigeria	[12]
	*Arachis hypogea* L.	peanuts	NA	Leaves	Decoction	Oral	Skin	Ashanti	Ghana	[26]
	*Aspalathus linearis* (Burm.f.) R.Dahlgr.	Rooibos	Inkanga	leaves	Leaves boiled in water	Taken orally as tea	Cervical	Eastern Cape	South Africa.	[31]
	*Baphia nitida* Lodd.	Camwood, barwood, African sandalwood	NA	Leaves	Decoction	Orally	Breast, skin, prostate, stomach, brain, throat	Ashanti	Ghana	[26]
	*Burkea africana* Hook.	Wild seringa	Umnondo, mukarati	Roots and stem back	Decoction	orally	Blood, colon	Matebelele land	Zimbabwe	[24]
	*Caesalpinia benthamiana* (Baill.) Herend. & Zarucchi	Tiger’s claw	NA	Leaves	Cold maceration, decoction, wound dressing	Orally, topically	Liver	Ashanti	Ghana	[26]
	*Caesalpinia bonduc* (L.) Roxb	Grey nicker, nicker bean, fever nut	NA	Roots	NA	Topically	Genital, prostate	Ashanti	Ghana	[26]
	*Cajanus cajan* (L.) Millsp.	Pigeon pea	Zikolimbo	Leaves, stem and root	Burnt	Topical, applied on infected skin	Breast	Elgon	Uganda	[23]
	*Calliandra portoricensis* (Jacq.) Benth.	Red powder puff	NA	Leaves	NA	NA	Prostate	NA	Nigeria	[12]
	*Calpurnia aurea*	Common calpurnia	Degeta	Leaves	Powdered leaves mixed with water	Topical	Neck	NA	Ethiopia	[20]
	*Cassia alata* L. Syn. *Senna alata* (L.) Roxb.	Candle bush, ringworm shrub	NA	Leaves	NA	NA	NA	NA	East and West Africa	[12]
	*Cassia sieberiana* DC.	Drumstick tree	Aridantooro (Yoruba)	Leaves	NA	NA	NA	NA	Nigeria	[12]
	*Castanospermum australe* A. Cunn & C. Fraser ex Hook	Moreton bay chestnut	NA	Leaves	NA	NA	Human drug-sensitive CCRF-CEM and multidrug-resistant CEM/ADR5000	NA	South Africa	[15]
	*Desmodium adscendens* (Sw.) DC.	Strongback, sweetheart, wild groundnut	NA	Leaves, stem	Tea, decoction	Oral	Prostate, breast, throat, brain	Ashanti	Ghana	[26]
	*Detarium microcarpum* Guill. & Perr.	sweet detar, tallow tree	Taura	Leaves, Bark, Root	Decoction, Infusion	Oral	NA	Mubi,	Adamawa, Nigeria.	[33]
	*Dialium dinklagei* Harms	Velvet tamarind		Bark, leaves	Decoction	Oral	Skin	Ashanti	Ghana	[26]
	*Dialium guineense* Willd.	Velvet tamarind	Tsamiyan biri	Leaves	Decoction and maceration	Oral	NA	Mubi	Adamawa Nigeria	[33]
	*Elephantorrhiza elephantina* (Burch.)	Eland’s bean, elephant’s root	Mupangara (Shona)	Leaves	NA	NA	Breast	All provinces excluding Mpumalanga	Southern Africa	[12]
	*Entada abyssinica* A.Rich.	Splinter bean	NA	Bark	Decoction	Oral	Breast	Ashanti	Ghana	[26]
	*Entada abyssinica* Steudel ex A. Rich	Tree entada	Kishembe	Leaves, stem & roots	Decoction	Oral	GIT, prostate	Elgon	Uganda	[23]
	*Enterolobium cyclocarpum* (Jacq.) Griesb.	Devil’s ear tree, monkey-ear tree	NA	Leaves	NA	NA	Liver	NA	West Africa	[12]
	*Erythrina abyssinica* Lam. Ex. DC	Lucky bean, flame tree	Kitugulu	Stem, root back	Decoction	Eaten raw, drunk	Cervical, oesophageal	Elgon	Uganda	[23]
	*Erythrina addisoniae* Hutch. & Dalziel	NA	NA	Leaves	NA	NA	Liver	NA	West Africa	[12]
	*Erythrina senegalensis* DC.	Senegal coral tree	NA	Whole plant	Nasal drops	NA	Head	Ashanti	Ghana	[26]
	*Erythrina senegalensis* DC.	Senegal coral tree	Gbengben tchikoloka	Root	Powdered	Topical	Throat wound	Tem	Togo	[11]
	*Erythrina sigmoidea* Hua	NA	NA	Leaves	NA	NA	Leukaemia, breast, colon, brain, Liver	NA	Cameroon, Chad	[12]
	*Erythrophleum suaveolens* (Guill. &. Perr.)	Ordeal tree, sasswood	NA	Leaves	NA	NA	Breast, colon, prostate, leukaemia	NA	Nigeria	[12]
	*Griffonia simplicifolia* (DC.) Baill.	Griffonia	Kwakuo-aboto	Roots	Decoction	Oral	Breast	Ashanti	Ghana	[26]
	*Mimosa pudica* L.	Sensitive plant, touch-me-not	NA	Leaves	grounded	Topical	Breast	Ashanti	Ghana	[26]
	*Neonotonia wightii* (Arn.) J.A. Lackey.	Perennial soybean	NA	Leaves, stem barks	NA	NA	NA	NA	Kenya	[12]
	*Parkia biglobosa* (Jacq.) G.Don	African locust bean	NA	Bark, roots, leaves	For massage	Topically	Breast	Ashanti	Ghana	[26]
	*Peltophorum sp.*	Shield-bearing	NA	Bark	powder	NA	Liver	Ashanti	Ghana	[26]
	*Pericopsis laxiflora* (Benth. ex Baker) Meeuwen	Satin wood	Kodolia	Leaves, root	Decoction	Oral	Bone, chronic wound	Tem	Togo	[11]
	*Prosopis africana* (Guill. & Perr.) Taub.	Iron tree	Kiriya	leaves	Decoction	Orally	NA	Mubi, Adamawa	Nigeria	[33]
	*Pterocarpus erinaceus* Poiret.	African kino tree, barwood	NA	Leaves	NA	NA	NA	NA	Nigeria	[12]
	*Pterocarpus santaliniodes* DC.	Red sandalwood	NA	Leaves	Decoction	Orally	Lungs	Ashanti	Ghana	[26]
	*Pseudarthria hookeria* Wight & Arn	Velvet bean, bug-catcher	Nakikofra	Leaves, stem & roots	Decoction	Oral and topical	Breast	Elgon	Uganda	[23]
	*Senna siamea* (Lam.) H. S. Irwin & Barneby	Kassod tree, Siamese cassa	Zanguerati	Leaves	Decoction	Oral	Lung	Ewè	Togo	[11]
	*Sutherlandia frutescens* L. R.Br.	Cancer bush	Umnwele	Leaves, flower and seed.	Decoction from all the plant parts.	Oral administration	DU-145 prostate cancer cells, MDA-MB-231 and MCF-7 breast cancer cells	NA	South Africa	[34]
	*Tamarindus indica* L.	Tamarind	Nidiè	Fruits	Powdered	Topical	Breast	Kabyè	Togo	[11]
	*Tamarindus indica* L.	Tamarind	Mukuwe	Fruits	Infusion or decoction	Oral	GIT, prostate	Elgon	Uganda	[23]
	*Tetrapleura tetraptera* (Schum. & Thonn.) Taub.	Aidan fruits	Uhio (Igbo)	Leaves, fruits	Infusion	oral	Leukaemia	NA	Nigeria	[12]
Gunneraceae	*Gunnera perpensa* L.	River pumpkin, wild rhubarb	Ighobo (Xhosa)	Rhizomes	Decoction, infusion	Oral	NA	Eastern cape	South Africa	[31]
Guttiferae	*Harungana madagascariensis* Lam. ex Poir.	Dragon’s blood tree, orange-milk tree		Leaves, fruits, stem bark	NA	NA	Colon, skin, breast	NA	Kenya	[12]
	*Hypericum riparium* A.Chev	Large-leaved curry-bush		Leaves, flowers	NA	NA	Gastric	NA	NA	[12]
Hyacinthaceae	*Drimia sphaerocephala* Baker	Round-head drimia	Hlare-sa-noko (Sesotho)	Leaves, bulbs	NA	NA	NA	Eastern Cape, KwaZulu-Natal	Southern Africa	[12]
	*Eucomis autumnalis* (Mill.) Chitt.	Pineapple flower	Umathunga	Bulbs	Decoction from warmed bulbs	Taken orally	NA	Eastern cape	South Africa	[31]
	*Ornithogalum caudatum* Aiton	False sea onion	Alèwa gabóu	Bulbs	Decoction	Body bath	Breast	Kabyè	Togo	[11]
	*Merwilla plumbea* [Lindl.] Speta	Dwarf blue squill	Umasixabane Ugontsana	Bulbs	Decoction from warmed bulbs	Taken orally	NA	Eastern cape	South Africa	[31]
Hydnoraceae	*Hydnora africana*		Mwoyogwemutaka	Root	Decoction, mixed with *Tamarindus indica* and *Cajanus Cajan*	Oral	Prostate, cervical	Elgon	Uganda	[23]
Hypoxidaceae	*Hypoxis argentea* Harv. Ex Baker	Small yellow stars	Inongwe	Corms	Decoction	Orally	NA	Eastern cape	South Africa	[31]
	*Hypoxis hemerocallidea* Fisch. and C.A. Mey.	Yellow star	Inkomfe, kaffertulp	Root corm	Infusion, coked with food and forming into paste	Taken orally and paste is applied topically	Prostate	NA	South Africa	[35]
	*Hypoxis hemerocallidea* Fisch. and C.A. Mey.	Yellow star	NA	Corms	Infusion	Oral	DU-145 prostate cancer cells, MDA-MB-231 and MCF-7 breast cancer cells, testicular tumours	NA	South Africa	[34]
	*Hypoxis hemerocallidea* Fish C. A. Mey	Yellow star	Mabondi Gemukyigona	Tuber	Decoction	Oral, topical	Prostate, cervical, breast colon, lung, GIT, skin intestinal uterine, oesophageal bone & bone cancers	Elgon	Uganda	[23]
	*Hypoxis hemerocallidea* (Fisch. & C.A.Mey.)	Yellow star	NA	Corms	NA	NA	Blood	Matebeleland north	Zimbabwe	[24]
	*Hypoxis obtusa* Burch. Ex Ker Gawl	Star lily	Inkomfe (Zulu)	Corms	NA	NA	NA	KwaZulu-Natal, Limpopo	South Africa	[12]
	*Hypoxis rigidula* var. *pilosissima* Baker	Silver-leaves, star-flower		Leaves, bulbs	NA	NA	breast MCF7, renal TK10 and melanoma UACC62	NA	South Africa	[12]
Iridaceae	*Gladiolus quartinianus* A. Rich	Parrot-beak gladiolus	NA	Whole plant	NA	NA	Leukaemia, breast, colon, brain	NA	Cameroon, Senegal, Ethiopia	[12]
Lamiaceae	*Clerodendrum capitatum* (Wild.) Schumach. & Thonn.	NA	NA	Bark, roots	Decoction, ground	Oral	Breast, skin	Ashanti	Ghana	[26]
	*Fuerstia africana* T.C.E.Fr.	NA	NA	Leaves, stem barks	NA	NA	Colon	NA	Kenya	[12]
	*Hoslundia opposita* Vahl	Orange bird berry, bird gooseberry	NA	Leaves, rot, sap	Tea, decoction	Oral	Lungs, brain, skin	Ashanti	Ghana	[26]
	*Hyptis pectinata* (L.) Poit	Comb bush mints	NA	Barks, rots	Decoction	Oral	Skin, breast, brain	Ashanti	Ghana	[26]
	*Leonotis leonurus* (L.) R.Br.	Wild dagga or lion’s ear	Imunyane, umcwili (Zulu)	Whole plant	Tea	Oral	Human drug-sensitive CCRF-CEM and multidrug-resistant CEM/ADR5000 leukaemia cells		South Africa	[15]
	*Leonotis nepetifolia* (L.) R.Br.	Klip dagga, Christmas candlestick	Namusiriri	Leaves, stem & roots	Crushed and taken raw (infusion)	Oral	Prostate, cervical, breast colon, lung, GIT, skin intestinal uterine, oesophageal bone & bone cancers	Elgon	Uganda	[23]
	*Ocimum basilicum* L.	Basil	Konzonzonga	Leaves	Powdered	Topical	Skin	Kabyè	Togo	[11]
	*Ocimum gratissimum* L.	Clove basil	NA	Leaves	Squeezed leaves	Topical	Skin, breast, prostate, stomach	Ashanti	Ghana	[26]
	*Ocimum gratissimum* L.	African basil	Azèou/Kabyè; Estro/Ewè	Leaves	Decoction, maceration, powdered	Oral, nasal	Lung, brain, breast	Kabyè, Ewè	Togo	[11]
	*Ocimum labiatum* (N.E.Br.0) A.J. Paton. LC. *formerly Orthsiphon labiatus*	Pink sage bush, shell bush	Pienksalie (Afikaans)	Aerial parts	NA	NA	NA	NA	Southern Africa	[12]
	*Ocimum viride* Wild.	African basil	NA	leaves	Crushed leaves for washing,	Topical	Skin, genital	Ashanti	Ghana	[26]
	*Orthosiphon serratus* (Schltr.) A.J.Paton	NA	Kleinskulphbos	NA	NA	NA	Human drug-sensitive CCRF-CEM and multidrug-resistant CEM/ADR5000	KwaZulu-Natal, Mpumalanga	South Africa	[15]
	*Plectranthus barbatus* (Andrews) Benth.	Wolly plectranthus	NA	NA	NA	NA	Human drug-sensitive CCRF-CEM and multidrug-resistant CEM/ADR5000 leukaemia cells	NA	South Africa	[15]
	*Plectranthus ciliatus* E. Mey. ex Benth.	Speckled spur-flower	Umsuthuza, lephele-phele	NA	NA	NA	Human drug-sensitive CCRF-CEM and multidrug-resistant CEM/ADR5000 leukaemia cells	NA	South Africa	[15]
	*Plectranthus cyaneus* Gurke	Spur-flower	Wobulaka (Tuliguuye)	Leaves, stem & roots	Crushed and taken (infusion) or boiled and drunk (decoction)	Oral, topical	Skin	Elgon	Uganda	[23]
	*Plectranthus verticillatus* (L.f.) Druce.	Swedish ivy, gossip spurflower	Skindersalie (Afrikaans)	Leaves, stems	NA	NA	Breast MCF7, renal TK10 and melanoma UACC62		South Africa	[12]
	*Salvia africana* L.	Blue African sage	Wildesalie (Afrikaans)	Aerial parts	NA	NA	NS	Cape provinces	South Africa	[12]
	*Salvia apiana* Jepson	White sage	NA	NA	NA	NA	Human drug-sensitive CCRF-CEM and multidrug-resistant CEM/ADR5000 leukaemia cells	NA	South Africa	[15]
	*Salvia coccinea* Buch’hoz ex Etl.	Scarlet sage, blood sage	NA	Leaves, stem barks	NA	NA	Breast, oesophageal, colon	NA	Kenya	[12]
	*Salvia miltiorrhiza* Bunge	Red sage, redroot sage	NA	Aerial parts	NA	NA	Prostate	NA	NA	[12]
	*Tetradenia riparia* (Hochst.) Codd,	Misty plume bush, ginger bush	Ibozane, iboza (Zulu)	Leaves, stems	Infusion	oral	Human drug-sensitive CCRF-CEM and multidrug-resistant CEM/ADR5000	NA	South Africa	[15]
	*Vitex fischeri* Gurke	NA	Nfulubwa, Ffulubwa	Leaves	Decoction	Oral	NA	NA	Uganda	[32]
Lauraceae	*Beilschmiedia acuta* Kosterm.	NA	NA	Barks, leaves	NA	NA	Leukaemia, breast, colon, brain	NA	Cameroon, Central African Republic	[12]
	*Persea americana* Mill.	Avocado tree	Pekedo	Leaves, seeds	Decoction	Oral	Skin	Elgon	Uganda	[23]
	*Laurus nobilis*	Bay tree, bay laurel	NA	Leaves	NA	NA	Human drug-sensitive CCRF-CEM and multidrug-resistant CEM/ADR5000 leukaemia cells	NA	South Africa	[15]
Liliaceae	*Gloriosa superba* L.	Flame lily, climbing lily	Etse lebona	Roots	Chewed	Oral, topical	Breast	NA	Ethiopia	[20]
Loganiaceae	*Anthocleista schweinfurthi* Gilg.	NA	NA	Stem barks, leaves	NA	NA	Leukaemia, colon, breast	NA	Tropical Africa	[12]
Loranthaceae	*Tapinanthus oleifolius* (J.C. Wendl.) Danser	NA	Gui rouge	Stem	Powder	Topical	Breast	NA	Togo	[11]
Malvaceae	*Adansonia digitata* L.	African baobab	Kuka	Leaves	Decoction	oral	NA	Mubi, Adamawa	Nigeria	[33]
	*Adansonia digitata* L.	NA	NA	Bark	Decoction	Oral	Stomach, breast	Ashanti	Ghana	[26]
	*Abelmoschus esculentus* (L.) Moench	Okra	NA	seeds	Grounded	Topically	Skin	Ashanti	Ghana	[26]
	*Abelmoschus esculentus* (L.) Moench	Okra	Otigo, Bamia	Fruits	Cooked	oral	Stomach, rectum and colon	Kampala	Uganda	[32]
	*Cola nitida* (Vent.) Schott & Endl	Kola nut, kola	NA	Bark	Decoction	Oral	Lungs, skin	Ashanti	Ghana	[26]
	*Glyphaea brevis* (Spreng.) Monach	Shortleaf globe mallow	Dabara	Leaves	Decoction	Oral	Brain, skin	Ashanti	Ghana	[26]
	*Gossypium arboretum* L.	Tree cotton	NA	Leaves	Decoction	Oral	Stomach, throat	Ashanti	Ghana	[26]
	*Hibiscus cannabinus* L.	NA	NA	Leaves, stems		Topical	Skin, breast	NA	West Africa	[12]
	*Hibiscus sabdariffa* L.	Roselle	NA	Calyces	Decoction	Orally	All cancer	Bulawayo	Zimbabwe	[24]
	*Hermannia depressa*	Doll’s roses	Seletjana	Leaves, roots	Crushed leaves	NA	NA	Eastern cape	South Africa	[31]
	*Malva verticillate* L. var. crispa L.	Chinese mallow, cluster mallow	Lut	NA	NA	NA	Neck	NA	Ethiopia	[20]
	*Mansonia altissima* (A.Chev) A.Chev	African walnut	NA	Bark, roots	Cold maceration, decoction	Orally, massage	Breast, skin	Ashanti	Ghana	[26]
	*Sida acuta* Burm.f.	Common wireweed	NA	Leaves, roots, whole plant	Decoction, squeezed juice, grounded	Oral	Skin, breast, colorectal	Ashanti	Ghana	[26]
	*Sida cordifolia* L.	Flannel weed, heart-leaf sida	NA	Leaves	Decoction	Oral	Lungs	Ashanti	Ghana	[26]
	*Sterculia tragacantha* Lindl	Gum tragacanth	NA	Bark	Decoction	NA	Breast	Ashanti	Ghana	[26]
	*Triplochiton scleroxylon* K.Schum	African whitewood	wawa	Stem bark, leaves, root bark	Decoction, dressing, ground as lump, squeezed leaves	Oral, topical	Skin, breast	Ashanti	Ghana	[26]
	*Triumfetta cordifolia* A. Rich	Cordleaf burbark, burweed	NA	Leaves, stem bark	Poultice	Topical	Skin, breast	Ashanti	Ghana	[26]
Meliaceae	*Azadirachta indica* A.Juss	Neem tree	NA	Leaves, stem bark, root bark, fruits	Decoction, paste, ground bark, ground fruits	Oral, topical	Skin, breast, bone	Ashanti	Ghana	[26]
	*Azadirachta indica* A. Juss.	Neem tree	Kini	Stem bark	Decoction	Oral	Chronic wound	Kabyè	Togo	[11]
	*Azadirachta indica*	Neem tree	Murabaine	Leaves, roots and stems	Decoction	Oral	Prostate, cervical, breast colon, lung, GIT, skin intestinal uterine, oesophageal bone & bone cancers	Elgon	Uganda	[23]
	*Carapa procera* DC.	African crabwood	NA	Bark	Decoction	Oral	Breast	Ashanti	Ghana	[26]
	*Ekebergia capensis* Sparrman.	Cape ash, dogplum	NA	Leaves, stem barks	NA	NA	Skin, throat, breast	NA	Kenya	[12]
	*Entandrophragma angolense* (Welw.) C.DC	NA	NA	Bark, roots, leaves, root bark	Decoction, tea	Oral	Prostate, skin, breast, throat, stomach	Ashanti	Ghana	[26]
	*Entandrophragma cylindricum* (Sprague) Sprague	Sapele, sapeli mahogany	NA	Leaves, bark	Decoction	Oral	Lung, skin	Ashanti	Ghana	[26]
	*Khaya anthotheca* (Welw.) C.DC.	East African mahogany	NA	Bark	Decoction	Oral	Breast, prostate	Ashanti	Ghana	[26]
	*Khaya ivorensis* A. Chev.	Lagos mahogany	NA	Leaves, stem bark	NA	NA	NA	NA	Nigeria, Southern Africa	[12]
	*Khaya senegalensis* (Desv.) A.Juss	African mahogany, khaya wood	NA	Bark, roots	Decoction, maceration, tincture, percolate, cream, tea	Oral, topical, massage	Stomach, skin, breast, prostate, lungs	Ashanti	Ghana	[26]
	*Khaya senegalensis* (Desr.) A. Juss.	Senegal mahogany	Hemou/Kabyè, Frimou/Tem	Stem back, leaves	Decoction, powdered, maceration	Oral, body bath, nasal	Chronic wound, skin, brain, bone	Kabyè, Tem	Togo	[11]
	*Lovoa* *trichilioides* Harms	African walnut, Congo wood	Musonko	Bark, seeds leaves,	Crushed and applied as an ointment	Oral	NA	NA	Uganda	[32]
	*Melia azedarach* L.	Chinaberry, syringa berry tree	NA	Leaves, stem barks	NA	NA	Colon, oesophagus	NA	Kenya	[12]
	*Pseudocedrela kotschyi* (Schweinf.) Harms	NA	Doutotorè	Root, leaves	Decoction, maceration	Oral, body bath	Breast and bone	Tem	Togo	[11]
	*Trichilia emetica* Vahl.	Natal mahogany	Umkhuhlu (isiXhosa)	Leaves, stem bark	NA	NA	Breast, skin, colon, prostate	NA	Tropical Africa	[12]
	*Turraea heterophylla* Sm	Honeysuckle tree	NA	Stem, leaves, roots, fruits	Mix with oil, decoction, ground leaves, tincture	Oral, massage	Stomach, prostate, joint, breast, liver, throat	Ashanti	Ghana	[26]
Melianthaceae	*Bersama abyssinica* Fresen. subsp. *abyssinica*	Winged bersama	Azamirr	Stem bark	Infusion	Oral	All cancer	NA	Ethiopia	[20]
	*Melianthus major* L.	Honey flower	Ubutyayi	Leaves	Decoction	Oral	NA	Eastern Cape	South Africa	[31]
Menispermaceae	*Cissampelos capensis* L.	Cape moonseed vine	Umayisake	Root	Paste	Topical	Skin and stomach	Eastern Cape	South Africa	[31]
	*Cissampelos mucronata* A.Rich.	NA	NA	Leaves	Decoction	Oral	Skin	Ashanti	Ghana	[26]
	*Tiliacora funifera* (Miers) Oliv.	NA	NA	Leaves	Decoction	Oral	Breast, throat	Ashanti	Ghana	[26]
Mimosaceae	*Dichrostachys cinerea* (L.) Wight and Arn.	NA	Sozossi	Root	Sauce	Oral	Breast	Tem	Togo	[11]
	*Parkia biglobosa* (Jacq.) R.Br. ex G.Don	NA	Soulou	Root, stem back	Decoction	Oral, body bath	Chronic wound and breast	Kabye	Togo	[11]
Moraceae	*Antiaris toxicaria* Lesch	False iroko, upas tree	NA	Bark	Decoction	Oral	Breast	Ashanti	Ghana	[26]
	*Dorstenia barnimiana* Schweinf.	NA	Worq bemeda	Roots, tuber	NA	Topical	Visible tumours	NA	Ethiopia	[20]
	*Ficus asperifolia* Miq.	NA	NA	Roots, bark, leaves, stem	Decoction, ground	Oral	Skin, breast, lung	Ashanti	Ghana	[26]
	*Ficus dawei* Hutch	NA	Muwo	Bark	Decoction	Oral	Breast	Mukono/Buikwe	Uganda	[32]
	*Ficus elatica* Roxb. Ex Hornem	Rubber fig	NA	Leaves, roots	Decoction (root), tea (leaves)	Oral	Stomach, prostate, lung	Ashanti	Ghana	[26]
	*Ficus exaperata* Vahl	Sandpaper tree, white fig	NA	Leaves, stem, bark	Sap	Topical	Breast	Ashanti	Ghana	[26]
	*Ficus natalensis subsp. leprieurii* (Miq.) Hochst. C.C.E	Barkcloth fig	NA	Bark	Decoction	Oral	Breast	Ashanti	Ghana	[26]
	*Ficus* *natalensis* Hochst.	Barkcloth fig	*Mugaire*	Roots	NA	NA	Cancerous wound	Iganga	Uganda	[32]
	*Milicia excelsa* (Welw.) C.C.Berg	African teak	NA	Bark, leaves	Ground, decoction	Oral	Skin, prostate	Ashanti	Ghana	[26]
	*Milicia regia* (A.Chev.) C.C.Berg	NA	Odum	Bark, leaves	Decoction, tea	Oral	Lungs, skin, stomach, throat, heart	Ashanti	Ghana	[26]
	*Morus mesozygia* Linnaeus	African mulberry		Leaves	NA	NA	Leukaemia, breast, colon, liver, brain	NA	Tropical Africa	[12]
Moringaceae	*Moringa oleifera* Lam.	Moringa	Moringa	Leaves, roots, seeds	Infusion, decoction, powder put in porridge	NA	All cancer	Masvingo	Zimbabwe	[24]
	*Moringa oleifera* Lam.	Moringa	Kpadadrè	Leaves, stems	Powdered	Oral	Breast	Kabye	Togo	[11]
	*Moringa* *oleifera* Lam.	Moringa	NA	Leaves, root, back, seeds	NA	NA	Prostate, lung, colon and rectal	Kampala	Uganda	[32]
Musaceae	*Musa acuminata* Colla	Dwarf Cavendish abab	Akori	NA	Powdered	Topical	Skin, chronic wound	Kabyè	Togo	[11]
	*Musa* × *paradisiaca* L.	NA	NA	Leaves, roots	Mashed leaves, decoction	Oral, topical	Stomach, throat, skin, breast	Ashanti	Ghana	[26]
Myrsinaceae	*Ardisia kivuensis* Taton	Coralberry	NA	NA	Leaves, fruits	NA	Breast, cervical	NA	NA	[12]
	*Myrsine africana* L.	African boxwood, Cape myrtle	Thakisa, sethakhisa (Sotho)	NA	Leaves	NA	Breast MCF7, renal TK10 and melanoma UACC62	NA	South Africa	[12]
	*Pycnanthus angolensis* (Welw.) Warb.	NA	NA	NA	Bark	Powdered, decoction	Topical, oral	Ashanti	Ghana	[26]
	*Rapanea melanophloeos* (L.) Mez.	Cape beech	isiQwane sehlati (Xhosa)	NA	Leaves	NA	Breast MCF7, renal TK10 and melanoma UACC62	NA	South Africa	[12]
Myrtaceae	*Psidium guajava* L.	Common guava, yellow or apple guava	NA	NA	Fruits, bark, leaves	Topical	Stomach, skin	Ashanti	Ghana	[26]
	*Syzygium cumini* (L.) Skeels	Malabar plum, java plum	Jambula	Leaves, roots, stem	Decoction, burnt	Oral, topical	Prostate, cervical, breast colon, lung, GIT, skin intestinal uterine, oesophageal bone & bone cancers	Elgon	Uganda	[23]
	*Syzygium guineense* (Willd.) DC.	Woodland waterberry		Leaves, stem barks	NA	Topical	Skin	NA	Kenya	[12]
	*Psidium guajava* L	Common guava, yellow or apple guava	Lipella	Leaves	Mixed with *Rhoicissus tridentata* (L.f.) Wild & R.B.Drumm.& *Combretum* *mole* G-don Drumm boiled	Oral	Prostate, cervical, breast colon, lung, GIT, skin intestinal uterine, oesophageal bone & bone cancers	Elgon	Uganda	[23]
Nephrolepidaceae	*Nephrolepsis exaltata* (L.) H.W. Schott.	Sword fern, Boston fern	NA	Leaves	NA	NA	Prostate	NA	South Africa	[12]
Nyctaginaceae	*Boerhavia diffusa* L.	NA	NA	Roots	Decoction	Oral	Brain, prostate	Ashanti	Ghana	[26]
Nyssaceae	*Camptotheca acuminata* Decne.	Happy tree, cancer tree		Leaves, stem bark	NA	NA	NA	NA	Southern, Eastern, Western and central Africa	[12]
Olaceae	*Olax subscorpioidea* Oliv.	NA	Ifon (Yoruba), Igbulu/Osaja (Igbo)	Leaves	NA	NA	NA	NA	Nigeria	[12]
	*Ximenia americana* L.	Hog plum, tallow wood	NA	Leaves	NA	NA	NA	NA	East Africa	[12]
Oleaceae	*Olea capensis* L.	Small or black ironwood	NA	Leaves, stem barks	NA	NA	Skin	NA	Kenya	[12]
	*Olea hochstetteri* Baker	East African olive wood	NA	Leaves stem barks	NA	NA	NA	NA	Kenya	[12]
Opiliaceae	*Opilia amentacea* Roxb.	NA	Kalibinou	Roots	Decoction	Oral	Lung	Kabye	Togo	[11]
Oxalidaceae	*Oxalis corniculata* L.	NA	Kajjampuni, Kaanhunu	Leaves	Ponded, crushed	Topical	Skin, uterine	Mukono/ Buikwe, Iganga	Uganda	[32]
Pandaceae	*Microdesmis puberula* Hook.f. ex Planch.	NA	NA	Fruits	Ground fruits	Topical	Breast	Ashanti	Ghana	[26]
Papaveraceae	*Argemone mexicana* L.	Mexican prickly poppy, flowering thistle	NA	Leaves	Decoction	Oral	Throat, breast	Ashanti	Ghana	[26]
Passifloraceae	*Adenia cissampeloides* (Planch. ex Hook) Harms	Snake climber	NA	Leaves	Decoction	Topical	Lungs	Ashanti	Ghana	[26]
	*Adenia lobata* (Jacq.) Engl.	NA	NA	Bark, roots, leaves	Decoction, ground	Topical	Breast, skin	Ashanti	Ashanti	[26]
	*Passiflora edulis* Sims	Passion fruits	NA	Leaves, fruits	NA	NA	Leukaemia		Central and East Africa, Cameroon.	[12]
Pedaliaceae	*Sesamum indicum* L.	Sesame	Goussi	Fruits	Sauce	Oral	Breast	Ewè	Togo	[11]
Phyllanthaceae	*Bridelia micrantha (Hochst.) Bail*	Coastal golden-leaf	Kigakala	Leaves, roots and stems	Boiled & drunk (decoction) and applied in infected skin	Oral, topical	Skin, prostate, cervical	Elgon	Uganda	[23]
	*Phyllanthus fischeri* Pax	NA	NA	Leaves, fruits, stem bark	NA	NA	NA	NA	Kenya	[12]
	*Phyllanthus fratenus* G. L.Webster	Kudu berry	NA	Bark	Decoction	Topical	Skin	Ashanti	Ghana	[11]
	*Pseudolachnostylis maprouneifolia* Pax (Agg)	NA	Mutsonzwa, mukurazviyo	Leaves	Infusion instilled into affected eyes, decoction	Oral	Eye, skin	Manica land	Zimbabwe	[24]
	*Uapaca togoensis* Pax	NA	NA	Leaves, fruits, stem bark	NA	NA	Leukaemia, breast, colon		Tropical Africa	[12]
Piperaceae	*Piper capense* L.f.	Wild pepper, Ethiopian long pepper	NA	Leaves, fruits	NA	NA	Leukaemia, breast, colon		Guinea, Ethiopia, South Africa	[12]
	*Piper guineense* Schumach. & Thonn.	False cubeb, Guinea cubeb	Atigali	Fruits	Decoction	Oral	Chronic wound	Kabyè	Togo	[11]
	*Piper umbellatum* L.	Cow-foot leaf	NA	NA	NA	NA	NA	Ashanti	Ghana	[26]
Pittosporaceae	*Pittosporum viridiflorum* Sims	Cheese wood, white cape beech	Umgqwengqwe	Bark, root	Infusions	Oral	NA	Eastern Cape	South Africa	[31]
Plantaginaceae	*Scoparia dulcis* L.	NA	NA	Roots, bark, leaves	Ground roots, decoction	Oral, topical	Breast, skin	Ashanti	Ghana	[26]
Plumbaginaceae	*Plumbago zeylanica* L.	Ceylon plumbago	Amerra	Leaves	Juice from fresh leaves	Oral	NA	NA	Ethiopia	[20]
Poaceae	*Bambusa vulgaris* Schrad.	Common bamboo	NA	Leaves	Decoction	Oral	Stomach	Ashanti	Ghana	[26]
	*Brachyachne obtusiflora* (Benth.) C.E.Hubb.	NA	NA	Roots	NA	Topical	Skin, genital	Ashanti	Ghana	[26]
	*Cymbopogon citratus* (D.C.) Stapf.	Lemon grass	NA	Leaves	NA	NA	Colon	NA	Kenya	[12]
	*Cymbopogon citratus* (DC) Stapf	NA	Akisube, Kisubi	Leaves	Decoction	Oral	NA	Pallisa	Uganda	[32]
	*Eleusine indica* (L.) Gaertn.	Indian goosegrass, crowfoot grass	Adandala	Root	Powdered	Topical	Breast	Tem	Togo	[11]
	*Imperata cylindrica* (L.) Raeusch.	Cogon, cottonwool grass	NA	Leaves	NA	NA	Leukaemia, breast, colon, brain	NA	West and East Africa	[12]
	*Zea mays* L.	Corn	NA	Com, grains, leaves	Ground grains as poultice	Topical	Skin	Ashanti	Ghana	[26]
	*Zea mays* L.	Corn	Samiriè	Stem	Powdered	Oral	Lung, chronic wound	Kabyè	Togo	[11]
	*Sporobolus pyramidalis* P. Beauv.	Cat’s tail grass	Faux gazon	Roots, fruits	Powdered	Topical	Breast, chronic wound	NA	Togo	[11]
Polygalaceae	*Securidaca sp.*	Hatchet	NA	Bark	Cream	Topical	Breast	Ashanti	Ghana	[26]
	*Securidaca longepedunculata* Fresen.	Violet or fibre tree	Fozi	Root	Maceration, powder,	Body bath, nasal, topical	Breast, bone, brain	Tem	Togo	[11]
Polygonaceae	*Rumex usambarensis* (Engl. ex Dammer)	NA	Nankombi	Leaves, root, stems	Decoction	Oral	Prostate, cervical, breast colon, lung, GIT, skin intestinal uterine, oesophageal bone & bone cancers	Elgon	Uganda	[23]
Portulacaceae	*Portulaca oleracea* L.	NA	NA	Roots, leaves	ground on stone plaster as dressing, decoction	Topical, oral	Prostate, skin, throat, breast	Ashanti	Ghana	[26]
Primulaceae	*Maesa lanceolata* Forssk.	False assegai	Kinywabanji	Leaves, root, stem	Decoction	Oral	Skin	Elgon	Uganda	[23]
Ranunculaceae	*Aconitum napellus* L.	Aconite, Venus’ chariot	NA	Leaves	NA	NA	NA	NA	Southern Africa	[12]
	*Knowltonia capensis* (L.) Huth	Blistering leaves	Brandblare (Afrikaans)	Leaves	Crushed leaves are prepared as poultice	Topical	Skin	Eastern Cape	South Africa	[31]
Rhamnaceae	*Gouania longispicata* Engl.	NA	Namayendeyende	Leaves	Decoction, burnt leaves	Oral, topical	Skin	Elgon	Uganda	[23]
Rosaceae	*Prunus africana*	The African cherry	NA	Stem bark	Decoction	oral	prostate	NA	Cameroon, Equatorial Guinea	[36]
	*Prunus africana*	The African cherry	Tikur enchet	Roots	NA	NA	All cancer		Ethiopia	[20]
	*Prunus* *africana* (Hook.f.) Kalkman *(Pygeum* *africanum)*	The African cherry	Ngwabuzito, Ntaseesa,	Leaves, back	Decoction	Oral	Prostate	Mukono/ Buikwe,	Uganda	[32]
	*Prunus persica* (L.) Batsch	Peach	Mupichisi	Seeds, stem bark	Decoction and infusion	Oral	Skin	Matebeleland North	Zimbabwe	[24]
Rubiaceae	*Bertiera racemosa* (G.Don) K.Schum		Kakadua	Bark, leaves	Decoction	Oral	Breast, skin	Ashanti	Ghana	[26]
	*Coffea arabica* L.	Arabica coffee	Emwanyi	Leaves, fruits	Mixed with *Mangifera indica* and *Deinbollia fulva* *-fomentalla* Bak.f boiled, infusion	Oral	Prostate, cervical	Elgon	Uganda	[23]
	*Corynanthe pachyceras* K.Schum	NA	NA	Leaves	Tea	Oral	Stomach	Ashanti	Ghana	[26]
	*Crosspteryx febrifuga* (Afzel. ex G.Don) Benth.	Common crown-berry, crystal-bark	NA	Bark	Decoction	Oral	Prostate	Ashanti	Ghana	[26]
	*Fadogia agrestis* Schweinf. ex Hiern	Black aphrodisiac	Djangadjanga	Roots	Decoction	Oral	Liver	Tem	Togo	[11]
	*Gardenia brighamii* H. Mann	Forest gardenia	NA	Leaves	NA	NA	Human drug-sensitive CCRF-CEM and multidrug-resistant CEM/ADR5000	NA	South Africa	[15]
	*Gardenia ternifolia* Schumach. & Thonn.	Powder-bark gardenia	Kao	roots	Decoction	Oral	Breast	Kabyè	Togo	[11]
	*Morinda citrifolia* L.	Rotten cheese fruits	Noni	Leaves	Powdered	Oral	Breast	NA	Togo	[11]
	*Nauclea pobeguinii* (Pobeg.) Merr.	NA	NA	Leaves, fruits	NA	NA	Leukaemia, breast, colon, brain	NA	South, West and Centra tropical Africa	[12]
	*Nauclea latifolia* Sm	African peach	NA	Leaves, fruits	NA	NA	Leukaemia, breast, colon	NA	West tropical Africa	[12]
	*Neolamarckia cadamba* (Roxb.) Boser	Burflower tree	Kadam	Leaves	Decoction	Oral	NA	Mubi	Adamawa, Nigeria	[33]
	*Pachystigma pygmaeum* (Schltr.) Robyns	Dwarf crowned-medlar	Witappeltjie (Afrikaans)	Leaves	Decoction	Oral	Human drug-sensitive CCRF-CEM and multidrug-resistant CEM/ADR5000	NA	South Africa	[15]
	*Pausinystalia yohimbe* (K.Schum.) Pierre ex Beille	Yohimbe	Burantashi	Leaves, fruits	NA	NA	NA	NA	West Africa	[12]
	*Pavetta abyssinica* Fresen.	NA	NA	Leaves	NA	NA	NA	NA	Kenya	[12]
	*Psydrax schimperiana* (A. Rich)	NA	NA	Leaves	NA	NA	NA	NA	Kenya	[12]
	*Rubia cordifolia.* Linn	Indian madder	Kizambazambe	Leaves, root, stem	Mixed with *Ribia cordifera*. & *Gouania longispicata* Engl. Linn, boiled	Oral	Lung, skin	Elgon	Uganda	[23]
	*Rubus discolor* Weihe & Nees	Himalayan blackberry	Encheber	Roots	NA	NA	All cancer	NA	Ethiopia	[20]
	*Sarcocephalus latifolius* (Sm.) E. A. Bruce	African peach	Kitchatchalou	Roots, stemback	Powdered	Oral, topical	Lung, chronic wound	Tem	Togo	[11]
	*Spermacoce princeae* (K.Schum.) Verdc.	Button-weed	NA	Leaves	NA	NA	NA	NA	Kenya	[12]
	*Zanthoxylum zanthoxyloides* L.	NA	Kalao	Root back	Powdered, sauce	Topical, oral	Breast	Kabye	Togo	[11]
Rutaceae	*Afraegle paniculata* (Schumach.) Engl.	Nigerian powder-flask fruits	Ngone	Seed	Powder	Oral	Breast	Kabyè	Togo	[11]
	*Araliopsis synopsis* Engl.	NA	NA	Stem barks	NA	NA	Prostate	NA	Cameroon	[12]
	*Citrus aurantiifolia* (Christm.) Swingle	Key lime or acid lime	NA	Fruits, leaves, bark	Juice mis with latex of alstonia boonei for plaster, squeezed, boiled bark	Oral, topical,	Breast, skin, throat	Ashanti	Ghana	[26]
	*Citrus limon* (L.) Osbeck	Lemon	NA	Fruits	Juice	Oral	Breast, prostate	Ashanti	Ghana	[26]
	*Citrus limon* (L.) Burm. f	Lemon	Gnami	Fruits	Maceration	Oral	Lung, breast	Kabyè	Togo	[11]
	*Citrus reticulata* Blanco	Mandarin orange	Omuqugwa, Amacunga	Root	NA	NA	NA	Pallisa	Uganda	[32]
	*Citrus sinensis* (L.) Osbeck	Sweet oranges	NA	Fruits, leaves	Juice, decoction	Oral, wash	Cervical, brain, throat, prostate, stomach	Ashanti	Ghana	[26]
	*Fagaropsis angolensis* (Engl.) Dale	NA	mukuriampungu	Stem bark, leaves, root	Decoction, boiled, maceration, ash powder, grinded to powder.	Chewed or decoction is drunk	NA	Uganda	East Africa	[37]
	*Vepris soyauxii* (Engl.) Mziray	NA	NA	Leaves	NA	NA	Leukaemia, breast, colon, brain, liver	NA	West Africa	[12]
	*Zanthoxylum chalybeum* Engl.	Lemon scented knob wood	Agodaman, Rukuts	Roots	Pounded and added water	Oral	Cervical	Mukono/ Buikwe	Uganda	[32]
	*Zanthoxylum gilleti* (De Wild.) P.G.Waterman	East African satinwood	NA	Bark	Cold maceration	Oral	Liver	Ashanti	Ghana	[26]
	*Zanthoxylum usambarense* (Engl.).	NA	NA	Leaves, roots	NA	NA	Breast	NA	East tropical Africa	[12]
	*Zanthoxylum zanthoxyloides* (Lam.) Zepern. & Timler	Senegal prickly-ash, artar root	NA	Stem bark, root bark	Decoction	Oral	Stomach, skin, brain, breast	Ashanti	Ghana	[26]
	*Zanthoxylum zanthoxyloides* L.	Artar root	Kalao	Root back	Powdered, sauce	Oral, topical	Breast	Kabyè	Togo	[11]
Salicaceae	*Oncoba spinosa* Forssk.	Snuff-box tree	NA	Bark	Decoction	Oral	Skin	Ashanti	Ghana	[26]
	*Sideroxylon obtusifolium* (Humb. ex Roem.) Penn.	NA	NA	Leaves, fruits	NA	Oral	Prostate		Southern Africa	[12]
	*Trimeria grandifolia* (Hochst.) Warb.	Wild-mulberry, big-leaf trimeria		Leaves, fruits	NA	Oral	Prostate		South Africa	[12]
Sapindaceae	*Allophylus abyssinicus* (Hochst.) Radlk.	Forest velvet false-currant	Zipelele	Leaves, root, stem	Decoction	Oral	Lung	Elgon	Uganda	[23]
	*Blighia sapida* K.D.Koenig	Ackee apple	NA	Roots, bark, leaves, fruits, root bark	Poultice, decoction, tea, maceration, raw fruits	Oral	Lungs, breast, stomach, colorectal, skin	Ashanti	Ghana	[26]
	*Blighia sapida* K. D. Koenig	Ackee apple	Kpizou	Leaves, fruits	Decoction, sauce, powdered	Oral, topical	Chronic wound, lung, skin, breast, brain	NA	Togo	[11]
	*Blighia unijugata* Baker	Triangle-tops	NA	Leaves	Decoction	Oral	Breast, throat	Ashanti	Ghana	[26]
	*Blighia unijugate* Baker	NA	Nkuzanyana	Bark	Decoction	Oral	Cervical	Mukono/ Buikwe	Uganda	[32]
	*Deinbollia fulvotomentella* Baker f.	NA	Kifuti	Fruits, leaves	Decoction	Oral, topical	Prostate, cervical, breast colon, lung, GIT, skin intestinal uterine, oesophageal bone & bone	Elgon	Uganda	[23]
	*Paullinia pinnata* L.	Barbasco	Tuantini (Twi)	Climbers, stem	Decoction, cold maceration	Oral	Stomach, skin, liver, breast	Ashanti	Ghana	[26]
	*Paullinia pinnata* L.	Barbasco	Adji kpizou	Leaves, root	Decoction, powdered	Oral, root	Chronic wound, bone	Kabyè	Togo	[11]
Sapotaceae	*Synsepalum cerasifera* (Welw.) T.D.Penn.	NA	NA	Leaves, fruits	NA	NA	NA		Kenya	[12]
	*Tridesmostemon omphalocarpoides* Engl.	NA	NA	Stem barks	NA	Oral	Leukaemia, brain, breast		Cameroon, Gabon, Congo, DR Congo	[12]
	*Vitellaria paradoxa* C. F. Gaertn.	Shea tree	Woussa	Stem back, root	Decoction, sauce	Oral	Breast, chronic wound, brain	Tem	Togo	[11]
Simaroubaceae	*Brucea antidysenterica* J.F. Mill	NA	Waginos/aballo	Leaves, twigs	Pastes using powdered leaves or twigs	Topical	All cancer	NA	Ethiopia	[20]
	*Harrisonia abyssinica* Oliv.	NA	Netu (Nasambu or Nefulo	Leaves, roots, stem	Mixed with *Gouania longispicat* a Engl. & *Lantana trifolia* boiled	Oral	Prostate, cervical, breast, colon, lung, GIT, skin, intestinal, uterine, oesophageal bone & bone	Elgon	Uganda	[23]
Solanaceae	*Capsicum annum* L.	Paprika, chili pepper	NA	Leaves	Decoction	Oral	Throat	NA	Ghana	[26]
	*Capsicum frutescens* L.	Wild chili pepper	NA	Leaves	Decoction	Oral	Breast	NA	Ghana	[26]
	*Capsicum frutescens* L.	Wild chili pepper	Kamulali, Kamularu	Fruits	As food condiment	Oral	Prostate	Mukono/Buikw	Uganda	[32]
	*Lycopersicon esculentum* Mill.	Tomato	NA	Fruits, leaves, root	Extraction of juice from fruits, ground leaves, boiled roots	Oral, topical	Throat, lungs, prostate, breast, skin	Ashanti	Ghana	[26]
	*Nicotiana tabacum* L.	Cultivated tobacco	Taba	Leaves	Decoction	Oral	Chronic wound	Kabyè	Togo	[11]
	*Physalis angulata* L.	Ballon cherry, gooseberry	NA	Leaves		Massage	Breast	NA	Ghana	[26]
	*Physalis angulata* L.	Hogweed, gooseberry	Ribomboni	Stem back	Powdered	Oral	Chronic wound	Tem	Togo	[11]
	*Solanum aculeastrum* Dunal subsp. *aculeastrum*	Goat bitter-apple, poison apple	Itunga, Umthuma	Fruits and leaves	Decoction	Oral	Breast	Eastern cape	South Africa	[31]
	*Solanum erianthum* D. Don	Mullein nightshade, salvadora	NA	Leaves	NA	NA	Skin, lung	NA	NA	[12]
	*Solanum incanum* L.	thorn apple, bitter tomato	Nhundurwa	Fruits, rots, leaves	Fruits macerate, infusion and tincture	Macerate applied on affected area	Skin, breast, blood	Masvingo	Zimbabwe	[24]
	*Solanum mauritianum* Scop.	Ear leaf or woolly nightshade	NA	Leaves	NA	NA	NA	NA	Kenya	[12]
	*Solanum nigrum* L.	Black or blackberry nightshade	Embuayzerech embuay	Leaves, stems and roots	NA	NA	Cancerous sores	NA	Ethiopia	[20]
	*Solanum panduriforme* E. Mey	Bitter apple	NA	Whole plant	NA	NA	Breast MCF7, renal TK10 and melanoma UACC62	NA	South Africa	[12]
	*Solanum tormentosum* L.	Snake apple	Slangappel (Afrikaans)	Stems	NA	NA	Breast MCF7, renal TK10 and melanoma UACC62	NA	South Africa	[12]
	*Solanum torvum* Sw.	Turkey berry, devil’s fig susumber	NA	Fruits	Tea, soup	Oral	Stomach, breast	NA	Ghana	[26]
	*Solanum verbascifolium* L.	NA	NA	Leaves	Crushed leaves	Topical	Skin, genital, breast	Ashanti	Ghana	[26]
	*Withania somnifera* (L.) Dunal	Winter cherry	Gezawa	NA	NA	NA	NA	NA	Ethiopia	[20]
Sterculiaceae	*Cola nitida* (Vent.) Schott & Endl.	Kola nut	Coroo	NA	Powdered	Topical	Breast	Tem	Togo	[11]
	*Theobroma cacao* L.	Cacao tree	Cocoo	Leaves	Decoction	Topical	Chronic wound	Kabyè	Togo	[11]
	*Waltheria indica* L.	Sleepy morning	Fafouloumou	Roots	Decoction	Oral	Lung	Tem	Togo	[11]
Taxaceae	*Taxus baccata* L.	English or common yew	NA	Fruits, leaves	NA	NA	Breast, ovarian, lung	NA	Southern Africa	[12]
	*Taxus brevifolia* (Nuttall) Pilger	Pacific or western yew	NA	Fruits, leaves	NA	NA	Breast, ovarian, lung	NA	Southern Africa	[12]
Thymelaeaceae	*Gnidia involucrata* Steud. ex A.Rich.	NA	Mejrit, yezingero telba	Roots	NA	Oral	Breast	NA	Ethiopia	[20]
Urticaceae	*Urtica dioica* L	Common or stinging nettle	Nettle	Root tuber	Decoction, infusion	Oral	Skin	Elgon	Uganda	[23]
Verbenaceae	*Duranta erecta* L.	Golden dewdrop, pigeon berry	NA	Leaves, roots, bark, Fruits	NA	NA	All cancer	Masholand west	Zimbabwe	[24]
	*Lantana rugosa* (camara) Thunb.	Bird’s beer	Ubuhobe, mubanda	Leaves, roots	Decoction	Oral	Blood, skin	Matebeleland South	Zimbabwe	[24]
	*Lantana trifolia* L.	Ternate lantana	Namuserera	Leaves, root, stem	Decoction	Oral	Cervical	Elgon	Uganda	[23]
	*Stachytarpheta indica* (L.) Vahl	India snakeweed, Brazilian tea	NA	Leaves	Decoction	Oral/topical	Breast, skin	Ashanti	Ghana	[26]
Vitaceae	*Cyphostemma adenocaule* (A. Rich)	NA	Namakajo	NA	Mixed *Hypoxis hemerocallidea* Fish C. A. Mey. &Ave-Lall and *Psidium guajava* boiled	Oral, topical	Skin	Elgon	Uganda	[23]
	*Cyphostemma serpens* (Hochst. ex A.Rich.) Desc.		NA	Leaves	NA	NA	Cervical, skin, breast	NA	Kenya	[12]
	*Rhoicissus tridentata* (L.f.) Wild & R.B.Drumm.	Bitter grape, wild grape	Nakibondi	Leaves, tubers	Decoction, infusion	Oral	Prostate, breast	Elgon	Uganda	[23]
Xanthorrhoeaceae	*Aloe ferox* Mill	Bitter aloe, red aloe	Ikhala	Leaf sap, leaves, roots	Sap	topically	Skin	Eastern Cape	South Africa	[31]
Ximeniaceae	*Ximenia caffra* Sond.	Large sorplum	Munhengeni	Fruits, roots and seeds	Infusion, decoction, chewed	Oral	All cancer	Manicaland	Zimbabwe	[24]
Zingiberaceae	*Aframomum arundinaceum* (Oliv. & G.Hanb.) K.Schum	NA	NA	Leaves, fruits	NA	NA	Leukaemia, breast, brain	NA	Western and Central Africa	[12]
	*Aframomum melegueta* K.Schum.	Pepper coast, melegueta pepper	NA	Fruits, root	Poultice	Topical	Brain, stomach	Ashanti	Ghana	[26]
	*Aframomum melegueta* K. Schum.	Pepper coast, melegueta pepper	Colombo	Fruits	Powdered, decoction	Oral, topical, body bath	Throat, breast, bone, skin, chronic wound	Kabyè	Togo	[11]
	*Aframomum polyanthum* (K.Schum.)	NA	NA	Leaves, fruits	NA	NA	Leukaemia, breast, brain	NA	West Africa	[12]
	*Aframomum pruinosum* Gagnep.	NA	NA	Leaves, fruits	NA	NA	skin	NA	West Africa	[12]
	*Curcuma longa* L.	Turmeric	NA	Root	Infusion	Oral	NA	Eastern cape	South Africa	[31]
	*Curcuma longa* L.	Turmeric	Wissikoyè	Root	Powdered	Oral	Breast	NA	Togo	[11]
	*Zingiber officinale* Roscoe	Ginger	NA	Roots, rhizome	Ground	Oral, massage	Stomach, brain	Ashanti	Ghana	[26]
	*Zingiber officinale* Roscoe	Ginger	Wissikoe	Rhizomes	Powdered	Oral	Breast	Kabyè	Togo	[11]
	*Zingiber officinale* Roscoe	Ginger	NA	Rhizome, Leaves,	NA	Oral	Leukaemia, cervical, bile duct	NA	Tropical Africa, West Africa, Cameroon	[12]
	*Zingiber officinale* Roscoe	Ginger	Tangawunzi	Rhizome	Eaten raw, Mixed with Hypoxidaceae. *Hypoxis* and *Rhoicisuss* *tridentata,* boiled & drunk *hemerocallidea Fish* C. A. Mey. &Ave-Lall (Concoction)	Oral	Oesophageal	Elgon	Uganda	[23]
Zygophyllaceae	*Balanites aegyptiaca* (L.) Delile	Egyptian balsam, soap berry tree	NA	Bark	NA	Oral	Stomach	Ashanti	Ghana	[26]

NA Information not available.

## Data Availability

No new data were created or analysed in this study.
